# Sex differences in 5‐year incidence and prevalence of physical illnesses following early childhood autism diagnosis

**DOI:** 10.1002/jcv2.70108

**Published:** 2026-02-19

**Authors:** Yu‐Chieh Chuang, Yu‐Jui Huang, Meng‐Chuan Lai, Wen‐Yin Chen, Chun‐Hung Pan, Sheng‐Siang Su, Chian‐Jue Kuo

**Affiliations:** ^1^ Taipei City Psychiatric Center Taipei City Hospital Taipei Taiwan; ^2^ Department of Psychiatry School of Medicine College of Medicine Taipei Medical University Taipei Taiwan; ^3^ Psychiatric Research Center Taipei Medical University Hospital Taipei Taiwan; ^4^ Campbell Family Mental Health Research Institute Centre for Addiction and Mental Health Toronto Ontario Canada; ^5^ Department of Psychiatry Temerty Faculty of Medicine University of Toronto Toronto Ontario Canada; ^6^ Department of Psychiatry The Hospital for Sick Children Toronto Ontario Canada; ^7^ Department of Psychology Faculty of Arts and Science University of Toronto Toronto Ontario Canada; ^8^ Department of Psychiatry Autism Research Centre University of Cambridge Cambridge UK; ^9^ Department of Psychiatry National Taiwan University Hospital and College of Medicine Taipei Taiwan; ^10^ School of Medicine College of Medicine Fu Jen Catholic University New Taipei City Taiwan; ^11^ Department of Psychology National Chengchi University Taipei Taiwan

**Keywords:** autism, incidence, physical illnesses, prevalence, sex differences

## Abstract

**Background:**

Sex differences in the clinical presentation of autism are established, but evidence on early‐life co‐occurring physical illnesses in early‐diagnosed autistic individuals is scarce. This nationwide cohort study examined sex‐stratified incidence of physical illnesses within 5 years after autism spectrum disorder (ASD) diagnosis in children ≤5 years.

**Methods:**

Using Taiwan's National Health Insurance Research Database (2000–2019), we identified 36,656 autistic males and 9024 autistic females newly diagnosed before age 6 and followed for five years. Adjusted odds ratios and hazard ratios for physical illnesses were estimated via multivariate logistic and Cox regression, controlling for demographic, clinical, and healthcare utilization factors. Analyses were stratified by intellectual disability status.

**Results:**

Within 5 years following an ASD diagnosis, autistic females exhibited higher incidences of cardiovascular diseases, cerebrovascular diseases, epilepsy, and irritable bowel syndrome, with generally modest to moderate effect sizes (approximately 1.3–2.3). In contrast, autistic males consistently demonstrated a higher risk of asthma. Prevalence analyses revealed similar patterns. Stratified analyses further indicated that, among children without intellectual disabilities, autistic females remained at elevated risks for cardiovascular diseases and epilepsy, with effect sizes ranging from approximately 1.44 to 2.86. Additional increased risks of hyperlipidemia and atopic dermatitis were also observed in autistic females, whereas autistic males continued to show higher risks of asthma and diabetes mellitus.

**Conclusion:**

Autistic females in early childhood face greater cardiometabolic and neurological burdens, while males are more prone to respiratory and metabolic conditions. These sex‐ and cognition‐specific risk profiles support tailored screening—cardiovascular/metabolic monitoring for females and respiratory disease prevention for males—to inform clinical care and health policy.

## Key Points

What's known?

- Clinical sex differences in autism are recognized, but early‐life sex‐stratified patterns of physical illness remain unclear.

**What's new?**

- In a nationwide cohort of autistic children ≤5 years, females had higher risks of cardiovascular/cerebrovascular diseases, epilepsy, hyperlipidemia, and atopic dermatitis then males; males had higher asthma and diabetes risks than females.

What's relevant?

- These findings support sex‐ and cognition‐informed screening strategies in early childhood, including cardiovascular and metabolic monitoring for females and respiratory disease prevention for males, with implications for clinical practice and policy.

## INTRODUCTION

As an early‐onset, life‐long neurodevelopmental condition characterized by social communication differences and restricted and repetitive behaviors (Dawson et al., 2023; Zeidan et al.,^2022^.), the prevalence rates of autism have been found increasing worldwide, currently estimated at 32.2 per 1000 children, with males affected approximately 3.4 times more often than females (Shaw et al., 2025). Long under‐recognized and under‐served, autistic females' differences from autistic males on timing of diagnosis (McDonnell et al., 2021), clinical presentations (Edwards et al., 2024; Lai et al., 2022; Wood‐Downie et al., 2021), physical health (Kassee et al., 2020), and genetic background (Sandin et al., 2024) are finally getting increased clinical and research attention (Lai et al., 2023). Specifically, studies on sex differences of mortality found that autistic females face higher risks of all‐cause mortality than autistic males, warranting tailored clinical attention (Catalá‐López et al., 2022; Tsai et al., 2023).

Previous population‐based studies have examined sex‐stratified co‐occurring psychiatric illnesses of autistic individuals (Hsu et al., 2024; Martini et al., 2022), as well as sex‐linked modifiers in genetic studies (Rylaarsdam & Guemez‐Gamboa, 2019), suggesting that autistic females may exhibit a greater genetic load to manifest clinical autism (Warrier et al., 2022; Wigdor et al., 2022). Considering that autism share genetic predispositions with physical illnesses and congenital deformities (Havdahl et al., 2021; Jin et al., 2024; Kuo et al., 2022; Patel et al., 2022), it is important to examine sex differences in the risks of physical illnesses in autistic individuals from the earliest stage of life. The few studies evaluating co‐occurring physical conditions with autism separately by sex are all in adults (Liu et al., 2023; Ward et al., 2023). Investigating sex‐stratified physical illness risks in large, representative sample from the earliest stages of life—particularly among the subgroup of individuals with early‐diagnosed autism, which may reflect a higher underlying genetic load—offers much needed clinical and research insight. Specifically, examining incidence provides more clues for temporal relations, informing health promotion opportunities.

To address these knowledge gaps to guide health promotion from early years of life for autistic people, we examined sex differences of the prevalence and incidence of physical illnesses in autistic young children using a large‐scale, nationwide, population‐based cohort from Taiwan. The sample included preschoolers (aged ≤5 years) diagnosed with autism spectrum disorder (ASD) to compare the prevalence and incidence of physical illnesses between autistic male and female children in the 5 years after their initial ASD diagnoses. In addition, to align with language that is widely accepted within the autistic community, person‐first language was adopted throughout this manuscript when referring to the study population(Zajic & Gudknecht, 2024).

## METHODS

### Data sources

This study utilized data from Taiwan's National Health Insurance Research Database (NHIRD), covering the period between January 1, 1996, and December 31, 2022. Established in 1995, Taiwan's National Health Insurance (NHI) system provides near‐universal health coverage, serving approximately 23.3 million people or 99% of the population in Taiwan. The NHIRD encompasses a wide range of information, including beneficiary registration, medical claims, prescription records, and diagnostic codes based on the International Classification of Diseases, Ninth Revision, Clinical Modification (ICD‐9‐CM) or 10th Revision, Clinical Modification (ICD‐10‐CM). The NHIRD captures healthcare utilization across Taiwan's healthcare system, encompassing both inpatient and outpatient services. Importantly, outpatient claims include visits to hospital‐based outpatient departments as well as primary care clinics and community‐based practices. In Taiwan, ASD diagnoses are most commonly established in outpatient settings, including both hospital‐based services and primary healthcare, predominantly by pediatricians, psychiatrists, and rehabilitation physicians. To safeguard privacy, the NHI Administration encrypts all data that could identify patients or healthcare providers. This study was approved by the Research Ethics Committee of Taipei City Hospital, Taiwan (approval number: TCHIRB‐11206009‐E; the date of approval: June 19, 2023). Due to the retrospective design and deidentified nature of the data, the ethics committee exempted the study from requiring informed consent.

### Study cohort

**FIGURE 1:**
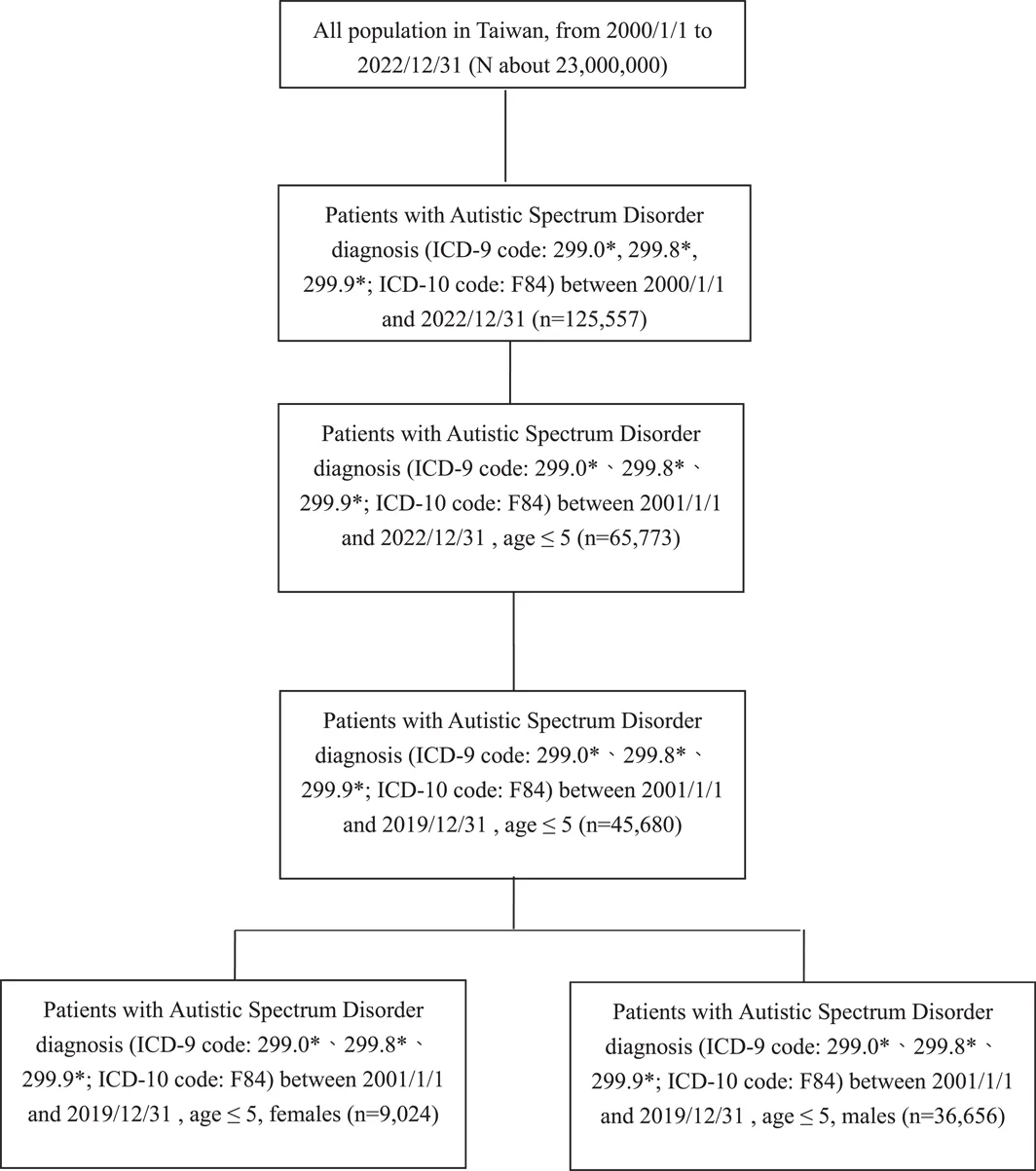
Study flow diagram. ICD‐9‐CM, International Classification of Diseases, Ninth Revision, Clinical Modification; ICD‐10‐CM, International Classification of Diseases, Tenth Revision, Clinical Modification codes.

Autistic children (aged ≤5 years) were identified from the NHIRD (Figure 1). A formal diagnosis of ASD (ICD‐9‐CM codes: 299.0, 299.8, 299.9; ICD‐10‐CM code: F84) was made by psychiatrists or pediatricians during outpatient visits or hospitalizations on at least one occasion before their sixth birthday, within the period from January 1, 2000, to December 31, 2019, which was defined as the index date of each included individuals. To ensure a cohort of newly diagnosed autistic children and reduce potential confounding from the COVID‐19 pandemic, individuals diagnosed with ASD before January 1, 2000, or after December 31, 2019, were excluded.

### Clinical and demographic variables associated with physical illnesses

Clinical and demographic data (as of the index date) were collected, including sex assigned at birth, age, urbanization level of residence, and parental monthly income (Table 1). Urbanization levels were categorized into five tiers in Taiwan (Liu, 2006): level 1 (highly urbanized), level 2 (moderately urbanized), level 3 (township), level 4 (sub‐rural), and level 5 (rural). Variables related to healthcare utilization within the 5 years following the ASD diagnosis included the frequency of hospitalizations, outpatient visits, and outpatient specialist visits (Table 2). Each category was further subdivided into psychiatric and non‐psychiatric hospital admissions or outpatient specialist visits.

**TABLE 1:** Demographic and clinical characteristics of autistic female and male children at the index date.

	Females (*N* = 9024)	Males (*N* = 36,656)	*p*‐value
	*n* (%)	*n* (%)	
Age, mean ± SD (years)	3.01 (1.26)	3.09 (1.24)	<0.001
1‐year‐old	1051 (11.65)	3182 (8.68)	<0.001
2‐year‐old	2536 (28.10)	10,648 (29.05)	
3‐year‐old	2144 (23.76)	8935 (24.38)	
4‐year‐old	1836 (20.35)	7496 (20.45)	
5‐year‐old	1457 (16.15)	6395 (17.45)	
Urbanization			0.152
Level 1	4891 (54.20)	19,603 (53.48)	
Level 2	2861 (31.70)	12,117 (33.06)	
Level 3	220 (2.44)	905 (2.47)	
Level 4	445 (4.93)	1749 (4.77)	
Level 5	517 (5.73)	1936 (5.28)	
Missing data	90 (1.00)	346 (0.94)	
Parental monthly income, (TWD^a^)			<0.001
0–20,479 (first quarter)	1867 (20.69)	6888 (18.79)	
20,480–25,599 (second quarter)	2295 (25.43)	9341 (25.48)	
25,600–44,799 (third quarter)	2698 (29.90)	10,619 (28.97)	
≧44,800 (fourth quarter)	2164 (23.98)	9808 (26.76)	

^a^ 31 TWD (Taiwan dollar) ≈1 US dollar.

**TABLE 2:** Healthcare utilization follow‐up for 5 years after the index date for autistic female and male children.

Characteristic *N* (%)	Females (*N* = 9024)	Males (*N* = 36,656)	*t* value	*p*‐value
	Mean (SD)	Mean (SD)		
Number of hospital admissions, *mean* (SD)	0.88 (2.29)	0.71 (1.90)	6.61	<0.001
Non‐psychiatric hospital admissions	0.84 (2.26)	0.64 (1.85)	7.49	<0.001
Psychiatric hospital admissions	0.05 (0.29)	0.07 (0.34)	−5.73	<0.001
Number of outpatient visits, *mean* (SD)	150.40 (88.41)	156.20 (88.17)	−5.62	<0.001
Non‐psychiatric outpatient visits	143.30 (85.48)	146.50 (84.68)	−3.19	0.001
Psychiatric outpatient visits	7.07 (12.23)	9.71 (14.22)	−17.78	<0.001
Specialist (number of outpatient visits)				
All	150.40 (88.41)	156.20 (88.17)	−5.62	<0.001
General medicine	1.63 (7.95)	2.02 (9.42)	−4.02	<0.001
Family practice	9.96 (19.37)	10.08 (19.68)	−0.53	0.599
Internal medicine	3.42 (10.41)	3.51 (10.50)	−0.77	0.442
Surgery	0.76 (3.10)	0.83 (2.89)	−1.93	0.054
Pediatrics	40.05 (37.60)	40.05 (39.10)	0.00	0.998
Gynecology	0.20 (1.14)	0.08 (1.42)	8.64	<0.001
Orthopedics	0.61 (1.91)	0.62 (2.00)	−0.35	0.730
Neurosurgery	0.08 (0.69)	0.06 (0.56)	1.92	0.054
Urology	0.09 (1.12)	0.26 (1.04)	−12.93	<0.001
Otorhinolaryngology	17.56 (26.87)	19.50 (29.02)	−6.03	<0.001
Ophthalmology	6.68 (7.85)	6.61 (7.50)	0.79	0.432
Dermatology	2.08 (4.28)	2.01 (4.36)	1.55	0.121
Neurology	0.11 (1.57)	0.07 (1.26)	2.20	0.028
Psychiatry	7.07 (12.23)	9.71 (14.22)	−17.78	<0.001
Rehabilitation	38.79 (44.91)	39.86 (45.61)	−2.01	0.045
Plastic surgery	0.15 (0.95)	0.14 (0.85)	1.50	0.133
Emergent department	1.76 (3.04)	1.92 (3.15)	−4.57	<0.001
Dentistry	11.60 (8.25)	11.43 (8.17)	1.77	0.077
Chinese herb medicine	7.77 (20.60)	7.45 (19.49)	1.35	0.176

### Physical illnesses

Physical illnesses were grouped by organ system into the following categories: cardiovascular diseases, cerebrovascular diseases, epilepsy, respiratory diseases, gastrointestinal diseases, endocrine diseases, and other diseases (e.g., atopic dermatitis, cancer, and HIV infection). The corresponding ICD‐9‐CM and ICD‐10‐CM codes are detailed in Supporting Information S1: Table S1.

The physical health conditions selected as outcomes were chosen based on their clinical significance, their relevance to pediatric health surveillance, and their reliable ascertainment within claims‐based data. Importantly, this outcome framework is consistent with prior population‐based studies, including our group's recent work using the same cohort (Chuang et al., 2025). The use of a consistent outcome framework facilitates comparability and cumulative interpretation across studies derived from the same cohort.

### Comparative analysis with the general population

To address the concern that observed sex differences may reflect background patterns present in the non‐autistic population, we conducted a comparative analysis using a 1:20 age‐ and sex‐matched general population cohort derived from the same database. Individuals with a diagnosis of ASD were excluded from the general population cohort.

The index date, outcome definitions, covariates, and statistical models were identical to those used in the primary autistic cohort analysis, allowing direct comparison of sex differences between autistic individuals and the general population (Supporting Information S1: Table S2).

### Statistical analysis

To provide a comprehensive assessment of co‐occurring physical conditions following autism diagnosis, we conducted both prevalence and incidence analyses. Prevalence analyses capture cross‐sectional disease burden, including congenital conditions and previously diagnosed physical illnesses present within the observation window, whereas incidence analyses focus on newly diagnosed conditions and clarify the temporal relationship between autism diagnosis and subsequent physical health outcomes. Presenting both measures allows a more complete characterization of early‐life medical burden that would not be fully captured by incidence analyses alone.

The prevalence of each physical illness within 5 year following the ASD diagnosis was analyzed. To compare prevalence rates of autistic females to males, a univariate conditional logistic regression model was applied, yielding unadjusted odds ratios. Additionally, Chi‐squared test and Student's *t* test were used to compare the demographic and clinical variables (except for sex), and healthcare utilization, between autistic males and females. A multivariate logistic regression model was constructed using backward stepwise selection to estimate adjusted odds ratios for 5‐year prevalence of physical illnesses, adjusted for demographic and clinical variables and healthcare utilization (i.e., number of outpatient visits and hospital admissions) (Table 3).

**TABLE 3:** 5‐year prevalence of co‐occurring physical illnesses in autistic female versus male children.

Characteristics	Females (*N* = 9024)	Males (*N* = 36,656)	Total (*N* = 45,680)	Adjusted odds ratio^a^	95% confidence interval	*p*‐value
5‐year after the index date						
Physical illnesses						
Cardiovascular diseases						
Hypertension	19 (0.21)	66 (0.18)	85 (0.19)	1.18	0.71–1.97	0.524
Ischemic heart disease	14 (0.16)	56 (0.15)	70 (0.15)	1.06	0.59–1.91	0.846
Other forms of heart disease	242 (2.68)	700 (1.91)	942 (2.06)	1.39	1.20–1.61	<0.001
Congestive heart failure	51 (0.57)	86 (0.23)	137 (0.30)	2.32	1.63–3.30	<0.001
Cerebrovascular diseases	151 (1.67)	427 (1.16)	578 (1.27)	1.42	1.18–1.72	<0.001
Epilepsy	954 (10.57)	1946 (5.31)	2900 (6.35)	2.15	1.97–2.34	<0.001
Respiratory diseases						
Pneumonia	3759 (41.66)	15,082 (41.14)	18,841 (41.25)	0.98	0.93–1.03	0.450
COPD	484 (5.36)	2469 (6.74)	2953 (6.46)	0.78	0.70–0.86	<0.001
Chronic bronchitis	204 (2.26)	860 (2.35)	1064 (2.33)	0.98	0.84–1.15	0.795
Asthma	2567 (28.45)	12,963 (35.36)	15,540 (34.02)	0.73	0.69–0.77	<0.001
Upper respiratory tract infection	5805 (64.33)	24,122 (65.81)	29,927 (65.51)	0.96	0.92–1.01	0.116
Gastrointestinal diseases						
Chronic hepatic disease	54 (0.60)	168 (0.46)	222 (0.49)	1.26	0.92–1.72	0.146
Ulcer disease	112 (1.24)	412 (1.12)	524 (1.15)	1.11	0.90–1.38	0.324
Endocrine diseases						
Diabetes mellitus	62 (0.69)	343 (0.94)	405 (0.89)	0.76	0.58–0.99	0.046
Others						
Diseases of arteries, arterioles, and capillaries	58 (0.64)	300 (0.82)	358 (0.78)	0.79	0.59–1.05	0.098
Diseases of veins and lymphatics, and other diseases of circulatory system	44 (0.49)	167 (0.46)	211 (0.46)	1.06	0.76–1.48	0.746
Cancer	118 (1.31)	462 (1.26)	580 (1.27)	1.00	0.81–1.23	0.974
Connective tissue disease	37 (0.41)	89 (0.24)	126 (0.28)	1.75	1.19–2.58	0.004
Renal failure	3 (0.03)	19 (0.05)	22 (0.05)	0.67	0.20–2.26	0.512
HIV Infection	–	–	–	–	–	–
Atopic dermatitis and related conditions	2032 (22.52)	7904 (21.56)	9936 (21.75)	1.06	1.00–1.13	0.035
Irritable bowel syndrome	140 (1.55)	563 (1.54)	703 (1.54)	1.03	0.85–1.24	0.780
Hyperlipidemia	30 (0.33)	63 (0.17)	93 (0.20)	1.95	1.26–3.02	0.003
Neurodevelopmental disorder						
Intellectual disabilities						
Mild	907 (10.05)	3229 (8.81)	4136 (9.05)	1.19	1.10–1.28	<0.001
Moderate	649 (7.19)	1994 (5.44)	2643 (5.79)	1.38	1.26–1.52	<0.001
>Severe	237 (2.63)	539 (1.47)	776 (1.70)	1.82	1.55–2.13	<0.001
Other/unspecified	1105 (12.25)	3473 (9.47)	4578 (10.02)	1.37	1.28–1.48	<0.001
Congenital deformities, chromosome abnormalities	1408 (15.60)	4493 (12.26)	5901 (12.92)	1.31	1.22–1.40	<0.001

^a^ Adjusted with demographic and clinical characteristics (except for sex) in Table 1, and numbers of outpatient visits and hospital admissions in Table 2.

The 5‐year incidence of each physical illness following an ASD diagnosis was assessed, with individuals with prior diagnoses excluded (Table 4). For example, individuals with a prior diagnosis of asthma were excluded from this calculation, and the incidence of asthma post‐ASD diagnosis was determined by dividing the number of new cases by the person‐years contributed by those without preexisting asthma in the cohort.

**TABLE 4:** 5‐year incidence of co‐occurring physical illnesses in autistic female versus male children.

Characteristics	Females	Males	Total *N*	Adjusted HR^a^	95% confidence interval	*p*‐value	FDR adjusted *P*‐value^b^
5‐year after the baseline							
Physical illnesses							
Cardiovascular diseases							
Hypertension	15 (0.17)	63 (0.17)	45,628	1.02	0.58–1.78	0.942	0.942
Ischemic heart disease	14 (0.16)	51 (0.14)	45,608	1.19	0.66–2.15	0.564	0.705
Other forms of heart disease	178 (2.04)	551 (1.54)	44,554	1.39	1.18–1.65	<0.001	<0.001
Congestive heart failure	29 (0.33)	47 (0.13)	45,362	2.53	1.60–4.00	<0.001	<0.001
Cerebrovascular diseases	105 (1.18)	329 (0.91)	45,122	1.34	1.08–1.68	0.009	0.025
Epilepsy	410 (4.94)	1077 (3.06)	43,522	1.76	1.57–1.97	<0.001	<0.001
Respiratory diseases							
Pneumonia	1863 (33.87)	7171 (33.30)	27,035	1.04	0.99–1.09	0.169	0.282
COPD	330 (3.91)	1842 (5.41)	42,483	0.93	0.85–1.01	<0.001	<0.001
Chronic bronchitis	160 (1.80)	742 (2.06)	44,945	0.92	0.78–1.09	0.335	0.529
Asthma	1654 (22.53)	7553 (27.16)	35,149	0.83	0.78–0.87	<0.001	<0.001
Upper respiratory tract infection	1631 (49.25)	6392 (49.73)	16,166	1.03	0.97–1.08	0.353	0.504
Gastrointestinal diseases							
Chronic hepatic disease	42 (0.47)	138 (0.38)	45,360	1.28	0.91–1.81	0.154	0.271
Ulcer disease	101 (1.13)	360 (0.99)	45,292	1.20	0.96–1.49	0.116	0.217
Endocrine diseases							
Diabetes mellitus	45 (0.50)	289 (0.79)	45,508	0.67	0.49–0.91	0.012	0.030
Others							
Diseases of arteries, arterioles, and capillaries	40 (0.45)	173 (0.48)	45,250	0.96	0.68–1.36	0.831	0.891
Diseases of veins and lymphatics, and other diseases of circulatory system	42 (0.47)	154 (0.42)	45,401	1.18	0.84–1.66	0.348	0.522
Cancer	79 (0.88)	300 (0.83)	45,248	1.12	0.88–1.44	0.363	0.495
Connective tissue disease	32 (0.36)	88 (0.24)	45,621	1.59	1.06–2.39	0.026	0.056
Renal failure	–	–	45,648	0.61	0.14–2.70	0.514	0.670
HIV Infection	–	–	45,677	‐	–	–	–
Atopic dermatitis and related conditions	700 (15.59)	2666 (15.00)	22,262	1.08	0.99–1.17	0.090	0.180
Irritable bowel syndrome	108 (1.22)	445 (1.24)	44,748	1.06	0.86–1.30	0.614	0.736
Hyperlipidemia	27 (0.30)	61 (0.17)	45,656	1.89	1.20–2.96	0.006	0.018
Neurodevelopmental disorder							
Intellectual disabilities							
Mild	737 (8.43)	2785 (7.76)	44,617	1.15	1.06–1.25	0.001	0.003
Moderate	523 (5.91)	1731 (4.78)	45,069	1.32	1.19–1.45	<0.001	<0.001
>Severe	206 (2.30)	481 (1.32)	45,525	1.85	1.57–2.18	<0.001	<0.001
Other/unspecified	850 (9.89)	2784 (7.86)	44,028	1.35	1.25–1.46	<0.001	<0.001
Congenital deformities, chromosome abnormalities	452 (6.72)	1990 (7.05)	34,951	0.98	0.88–1.09	0.679	0.783

^a^ Adjusted with demographic and clinical characteristics (except for sex) in Table 1, and numbers of outpatient visits, hospital admissions in Table 2.

^b^ Abbreviation: FDR, false discovery rate.

Using survival analysis with Cox regression model, hazard ratios (HRs) with 95% confidence intervals were computed by calculating the ratio of the incidence rate of a given physical illness of autistic females to its incidence rate of autistic males (Supporting Information S1: Table S3). To estimate adjusted hazard ratios (aHRs) with 95% confidence intervals for each physical illness, multivariate Cox regression models were constructed using backward stepwise selection; variables, including demographic and clinical factors (except for sex) and healthcare utilization (numbers of outpatient visits and hospital admissions), were retained in the final models (Table 4). The magnitude of risk for physical illnesses in autistic females compared to autistic males, as determined by the aHRs, was categorized as follows: mild (1–1.4), modest (1.41–1.7), moderate (1.71–3.0), and strong (3.01–8.0) (Chen et al., 2024). If aHR was less than 1 (indicating that the effect in males is larger than that in females), the reciprocal of these thresholds was calculated to categorize the magnitude of risk: mild (0.71–1), modest (0.6–0.7), moderate (0.33–0.59), and strong (<0.33).

To account for multiple comparisons across a large number of physical health outcomes, false discovery rate (FDR) control was applied using the Benjamini–Hochberg procedure. FDR‐adjusted *p* values were calculated, and an adjusted *p* value < 0.05 was considered statistically significant (see Table 4).

All statistical analyses were performed using SAS statistical software (version 9.4, SAS Institute, Cary, NC, USA), with statistical significance defined as a *p*‐value <0.01.

### Stratification analysis

We also conducted subgroup sensitivity analyses of autistic children with and without intellectual disabilities to assess the potential confounding effect (Supporting Information S1: Tables S4–S7 and Figures S1, S2).

### Sensitivity analysis

As a sensitivity analysis, Cox proportional hazards models were additionally constructed using birth date as the index date, with follow‐up beginning at birth and extending throughout the entire available follow‐up period (see Supporting Information S1: Tables S8, S9).

Also, to assess potential temporal changes in diagnostic practices, supplementary analyses compared baseline characteristics and healthcare utilization between autistic children diagnosed in January 1, 2001–December 31, 2009 and January 1, 2010–December 31, 2019 (see Supporting Information S1: Tables S10A, S10B).

## RESULTS

### Cohort characteristics

The clinical and demographic characteristics of the cohorts are summarized in Table 1. In the overall cohort, the male‐to‐female ratio was approximately 4.06. Both autistic males and females were diagnosed with ASD at a mean age of 3 years, with around 90% receiving their diagnosis between ages 2 and 5. Additionally, parental monthly income expenditure were significantly higher for autistic males than females.

Table 2 presents the healthcare utilization in autistic female versus male children over a 5‐year follow‐up period after ASD diagnosis. Overall, autistic females had a higher number of hospital admissions than autistic males; however, psychiatric hospital admissions were more common among autistic males. For outpatient visits, both non‐psychiatric and psychiatric visits were more frequent in autistic males than autistic females. Regarding specialist outpatient visits, autistic females had higher visit rates only in gynecology, while autistic males had more frequent visits across various specialties, particularly psychiatry.

### Prevalence of physical illnesses within 5 years after ASD diagnoses

Table 3 presents the prevalence rates of physical illnesses over 5 years after ASD diagnoses in autistic female versus male children. Autistic females had higher risks of various illnesses, including cardiovascular diseases (e.g., congestive heart failure), cerebrovascular diseases, epilepsy, and hyperlipidemia, compared to the autistic males. In contrast, autistic females exhibited a lower risk of asthma compared to autistic males. Additionally, autistic females showed no statistical significance for congenital deformities or chromosomal abnormalities comparing to autistic males.

### Incidence of physical illnesses within 5 years after ASD diagnoses

**FIGURE 2:**
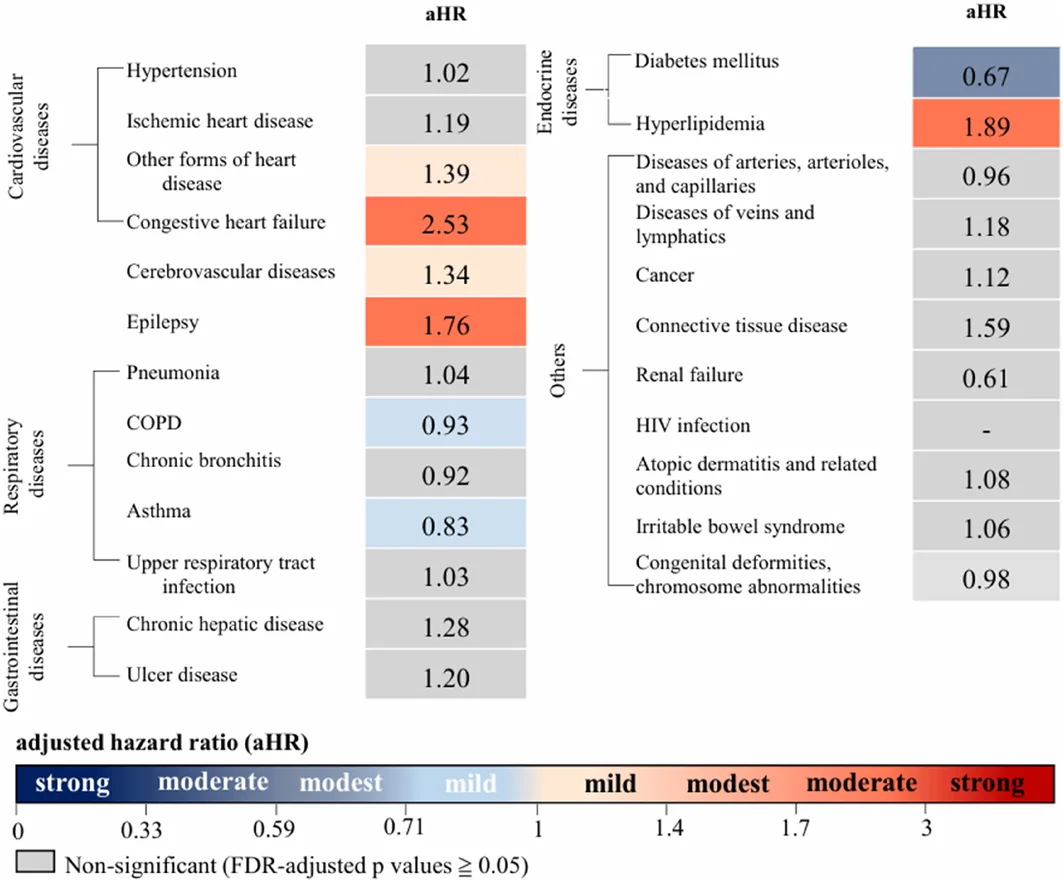
Adjusted hazard ratio of newly developed physical illnesses within 5 years after an initial autism spectrum disorder diagnosis in autistic females compared to autistic males.

Table 4 presents the incidence rates and aHRs for physical illnesses, comparing autistic females to autistic males over 5 years after ASD diagnoses. The aHRs were significantly elevated (indicative of higher risks in autistic females than males) for several cardiovascular diseases (e.g., congestive heart failure), cerebrovascular disease, epilepsy, and hyperlipidemia. The aHRs for congestive heart failure, epilepsy, and hyperlipidemia were all greater than 1.7, suggesting a moderate effect (Figure 2). Conversely, asthma and diabetes mellitus exhibited significantly lower aHRs (indicative of lower risks in autistic females than males) with mild effect and modest effect respectively. Notably, these associations remained statistically significant after FDR adjustment, with all FDR‐adjusted *p* values < 0.05 (Table 4).

### Comparative analysis with the general population

In the general population, sex‐stratified patterns differed substantially from those observed in the autistic cohort (Table S2). Females generally exhibited similar or lower risks than males for most neurodevelopmental and neurological conditions, including epilepsy and intellectual disability (ID). Lower risks among females were also observed for cerebrovascular disease and several respiratory and metabolic conditions, whereas higher risks were noted for hyperlipidemia, connective tissue disease, and renal failure.

When comparing sex‐stratified patterns between the autistic cohort and the general population, several conditions demonstrated contrasting associations. Notably, epilepsy and ID were associated with higher risks among autistic females but lower or comparable risks among females in the general population. Similarly, cerebrovascular disease showed an increased risk among autistic females, whereas females in the general population exhibited a reduced risk compared with males. Additionally, a statistically significant increased risk of cardiovascular diseases—including other forms of heart disease and congestive heart failure—was observed in autistic females but not in the general population. Conversely, autistic females showed a statistically significant lower risk of diabetes mellitus, whereas no such sex difference was detected in the general population. In contrast, sex differences for asthma, chronic obstructive pulmonary disease, and hyperlipidemia were largely consistent across both population.

Overall, sex differences with physical illnesses differed markedly between autistic individuals and the general population, with several conditions exhibiting population‐specific patterns.

### Stratification analysis

The stratification by the presence or absence of intellectual disabilities is shown in Table S4 and Table S6, in the context of a higher proportion of autistic females having intellectual disabilities than autistic males (Table 3). In autistic children without intellectual disabilities, significantly increased aHRs remained for other forms of heart diseases, congestive heart failure, epilepsy, and hyperlipidemia, indicating higher risks in autistic females than males. Additionally, atopic dermatitis showed a significant increase in aHRs. Cardiovascular diseases, epilepsy, and hyperlipidemia displayed modest to moderate effect size, with congestive heart failure exhibiting moderate effect (aHR 2.86). On the other hand, aside from asthma and COPD, which already showed a significantly decreased aHR in the full sample, diabetes mellitus also demonstrated a statistically significant reduction in aHR with a moderate effect, indicating lower risks in autistic females than males (Supporting Information S1: Figure S1).

In autistic children with intellectual disabilities, autistic females showed a statistically significant increase risks of incidence for epilepsy with a moderate effect, while autistic males exhibited a significantly higher risk of incidence for asthma with a mild effect (Supporting Information S1: Figure S2).

### Sensitivity analysis

In sensitivity analyses using birth date as the index date, the overall pattern of sex difference was largely consistent with the primary analysis based on diagnosis date. Autistic females continued to show higher risks than autistic males for epilepsy, ID across severity levels, and several cardiovascular outcomes, including other forms of heart disease and congestive heart failure. The magnitude of associations for some conditions differed modestly when follow‐up was initiated at birth. Specifically, effect estimates for epilepsy and selected cardiovascular diseases were amplified compared with the primary analysis, with the direction of associations remaining unchanged. In contrast, sex differences for respiratory conditions, including asthma and chronic obstructive pulmonary disease, remained stable across index‐date definitions, with autistic males consistently exhibiting higher risks than autistic females. No major reversals in sex differences were observed (see Supporting Information S1: Table S8).

When follow‐up was extended to the end of the study period, sex differences for several outcomes differed from those observed in the primary analysis. Autistic females continued to exhibit higher risks of epilepsy and ID across severity levels, while additional sex differences emerged for selected chronic conditions. Notably, autistic females demonstrated a higher cumulative risk of connective tissue disease compared with autistic males, an association that was not evident in the primary 5‐year post‐diagnosis analysis. In contrast, the sex differences for diabetes mellitus and hyperlipidemia observed in the primary analysis were attenuated and no longer statistically significant with extended follow‐up. For respiratory conditions, including asthma, autistic males consistently showed higher risks across both follow‐up definitions. Overall, extending the follow‐up duration revealed additional long‐term sex differences without reversing the direction of key neurological associations (see Supporting Information S1: Table S9).

To assess potential temporal changes in diagnostic practices, supplementary analyses compared baseline demographic characteristics and healthcare utilization between autistic children diagnosed in 2001–2009 and 2010–2019 (see Supporting Information S1: Tables S10A, S10B). Overall patterns were broadly comparable across diagnostic periods.

## DISCUSSION

This study offers total population‐based insights of early physical health in autistic children by directly examining sex differences in prevalence and incidence rates of physical illnesses in autistic children younger than 5 years of age. Within 5 years after being diagnosed with ASD, the prevalence of cardiovascular diseases, cerebrovascular diseases, epilepsy, atopic dermatitis and related conditions, and hyperlipidemia was significantly higher in autistic females than in autistic males. In contrast, autistic males had a higher prevalence of asthma. When further examining incidence (i.e., newly diagnosed illnesses), autistic females showed higher rates for cardiovascular diseases, cerebrovascular diseases, epilepsy, and hyperlipidemia than autistic males, with moderate effects observed for congestive heart failure, epilepsy, and hyperlipidemia; on the other hand, autistic males exhibited a higher rate for asthma, COPD, and diabetes mellitus, with a mild to modest effect. In autistic individuals without intellectual disabilities, significantly higher risks persisted for cardiovascular diseases and epilepsy in autistic females with additional observation for hyperlipidemia and atopic dermatitis and related conditions, with a modest to moderate effect; conversely, autistic males exhibited higher risks not only for asthma but also for diabetes mellitus. On the other hand, among autistic individuals with intellectual disabilities, autistic females exhibited a moderately increased risk of epilepsy, whereas autistic males demonstrated a mildly higher rate of asthma. Taken together, these findings indicate a consistent pattern of sex‐specific differences in early physical health among autistic children, with autistic females bearing a disproportionate burden of several neurological and cardiovascular conditions, even after accounting for co‐occurring intellectual disabilities.

Previous studies have conducted sex‐stratified analyses of co‐occurring physical illnesses, with the majority focusing on autistic adults (Dizitzer et al., 2020; Dubreucq et al., 2023; Kassee et al., 2020; Liu et al., 2023; Ward et al., 2023). Evidence indicates that autistic females face a nearly sevenfold increased risk of experiencing three or more co‐occurring physical health conditions (Dubreucq et al., 2023), particularly epilepsy, endocrine and reproductive problems (Kassee et al., 2020; Ward et al., 2023) as well as long‐term risks of cardiovascular and respiratory diseases (Liu et al., 2023). For children, although few studies have reported that autistic females in early childhood may be at higher risk for several conditions (e.g., hearing impairments, neurological disorders, epilepsy, metabolic abnormalities, gastrointestinal issues) compared to autistic males (Angell et al., 2021; Dizitzer et al., 2020), sex‐stratified analyses in this age group remain scarce, especially in areas such as endocrine, reproductive, and general physical health (Kassee et al., 2020). Taken together, these findings point to a substantial evidence gap in sex‐stratified physical health risks during early childhood, particularly among autistic children diagnosed early in life, limiting current understanding of early‐life disease burden and opportunities for timely health monitoring. Addressing this knowledge gap, our population‐based results indicated that autistic females exhibited higher risks of cardiovascular and cerebrovascular diseases, epilepsy, and irritable bowel syndrome compared to autistic males. Notably, several studies have highlighted the disadvantage in healthcare utilization among autistic females compared to autistic males, a pattern also observed in our cohort, along with the delayed diagnosis of ASD in autistic females (Lai et al., 2014; Nordahl, 2023; Ochoa‐Lubinoff et al., 2023; Rynkiewicz et al., 2024; Tint et al., 2023). These suggests that the risks of physical illnesses in autistic females may be still underestimated, particularly in the absence of appropriate population‐based reference frameworks to distinguish autism‐specific patterns from background sex differences.

By incorporating a comparative analysis with the general population, we were able to distinguish sex differences specific to autism from those reflecting background population patterns, thereby providing a novel framework for contextualizing sex‐stratified health disparities in autism. Several neurological conditions, including epilepsy and ID across severity levels, demonstrated contrasting sex‐stratified patterns between populations, with females in the general population showing lower or comparable risks, whereas autistic females consistently exhibited higher risks than autistic males.

A similar contrast was observed for selected cardiovascular outcomes: no significant sex differences were detected in the general population for other forms of heart disease or congestive heart failure, yet autistic females showed significantly elevated risks for these conditions. Together, these findings indicate that the observed sex differences cannot be explained solely by background population trends and suggest that autism status modifies sex‐related vulnerability to both neurodevelopmental and selected cardiovascular co‐occurring conditions. In contrast, sex differences for respiratory and metabolic conditions, such as asthma and hyperlipidemia, were largely consistent across both populations.

The associations between co‐occurring intellectual disabilities and both physical and mental health conditions in autistic individuals have been well established (Bishop‐Fitzpatrick & Rubenstein, 2019). Intellectual disabilities may alter autistic presentations, especially sex differences within autism (Saure et al., 2023). These phenotypic variations may contribute to the under‐identification or misdiagnosis of autistic females, potentially shaping both their clinical presentations and physical health outcomes. Additionally, there is disproportionate impact of co‐occurring intellectual disabilities on autistic females, who exhibit a higher risk of mortality compared to their male counterparts, with a mild to moderate effect (Catalá‐López et al., 2022; Hirvikoski et al., 2016; Huang et al., 2024). Building on this evidence, our findings further show that intellectual disabilities may be associated with different profiles of physical illnesses in autistic individuals during early childhood. In autistic children without intellectual disabilities, females demonstrated increased hyperlipidemia and atopic dermatitis on top of those found across the full sample. They also showed a decreased risk for diabetes mellitus. Notably, the elevated risks for cardiovascular disease and epilepsy, as well as the decreased risk for asthma, remained significant as found in the full sample. In contrast, autistic females with intellectual disabilities showed an increased risk for epilepsy and a decreased risk for asthma. These findings highlight the potential role of sex‐based and ID‐focused health screening for better early detection of physical illnesses, particularly in autistic females without intellectual disabilities who may tend to be overlooked in clinical practices (Lai et al., 2023).

To further examine whether these sex differences were influenced by analytic design, we conducted two additional sensitivity analyses using alternative index‐date definitions and follow‐up durations. Sensitivity analyses incorporating birth‐based index dates and extended follow‐up periods provided a life‐course perspective on disease occurrence. These analyses demonstrated that while effect sizes for certain conditions varied with follow‐up definition, the overall direction of key sex differences—particularly for neurodevelopmental disorders and epilepsy—remained consistent. This finding supports the robustness of the primary results and underscores the importance of considering cumulative disease burden beyond the post‐diagnostic period when evaluating sex differences in physical health among autistic individuals. In addition, although diagnostic practices evolved over time, supplementary analyses suggested that such temporal changes were unlikely to materially influence the main findings.

Females diagnosed with ASD, especially in early childhood, may exhibit more pronounced autistic traits compared to the broader group of autistic females (Wigdor et al., 2022). Overall, autistic females tend to carry a greater extent of genetic factors associated with autism compared to autistic males (Antaki et al., 2022; Rolland et al., 2023; Zhang et al., 2020), which is further related to co‐occurring intellectual disabilities (Warrier et al., 2022). This elevated genetic background—often involving de novo or protein‐truncating variants—has also been associated with physical illnesses across multiple organ systems (Jin et al., 2024; Kassee et al., 2020; Zhang et al., 2020). Notably, autism‐associated genes with pleiotropic effects, which may appear more frequently in females, have been linked to neurodevelopmental conditions and a broad range of physical illnesses (Kassee et al., 2020; Vorstman et al., 2017). Therefore, the higher prevalence of physical illnesses observed in autistic females may, at least in part, be attributable to their greater underlying genetic vulnerability. Meanwhile, it is an open question how non‐genetic biological factors (e.g., early environmental exposure, nutrition and intake) or socio‐contextual factors (e.g., family living situation) might contribute to the observed sex‐differential rates of physical illnesses in autistic young children.

### Strengths and limitations

To our knowledge, this study is the first to report sex‐stratified incidence of physical illnesses in autistic young children soon after an ASD diagnosis. Our findings provide new insights into the early physical health of autistic children, evidencing that sex differences in autism involve physical health emerging in early childhood. Using population‐based data, we constructed a nationally representative cohort of autistic children, minimizing selection bias. Additionally, the detailed diagnostic codes in the NHIRD allowed for comprehensive analyses of conditions across multiple organ systems.

Our study has some limitations. First, we did not intentionally match autistic males and females by age in order to maximize sample size and statistical power. Nevertheless, the mean age difference between sexes was minimal (3.01 vs. 3.09 years). Second, we relied exclusively on ICD‐9‐CM and ICD‐10‐CM codes for diagnoses, which may raise concerns about validity. The NHI Administration conducts annual audits to verify the accuracy of diagnostic codes in the NHIRD, assuring data integrity. Third, although we applied a washout period from January 1, 2000, to December 31, 2000 to exclude individuals who received an ASD diagnosis prior to the observation window, some cases of preexisting autism may have still been included. Additionally, the temporal sequence of illness onset may not perfectly capture the relationship between physical illnesses prior to January 1, 2000. Moreover, in the incidence analyses, the identification of individuals without prior physical disease relied on diagnostic records in administrative claims data, which may not accurately reflect true disease onset. Some physical conditions may have been present but undiagnosed or unrecorded before the observed autism diagnosis, and incomplete capture of healthcare encounters may further contribute to misclassification of pre‐existing conditions. Fourth, owing to the inherent limitations of the NHIRD, we did not have access to data on lifestyle factors, medication adherence, and laboratory tests, which could all affect the observed findings. Fifth, although the primary analyses focused on sex differences within the autistic population, supplementary analyses incorporating an age‐ and sex‐matched general population cohort indicated that several observed sex differences were specific to autism. Finally, this study focused on children diagnosed with autism in early childhood and therefore reflects health outcomes within an early‐diagnosed subgroup rather than the entire autistic population. Given well‐documented delays in autism diagnosis—particularly among females—our sex‐stratified estimates of physical illness risk, especially for females, may underestimate the true burden of physical health conditions in the broader autistic population and may not be fully generalizable to individuals diagnosed later in life.

### Future implications

Building on increasing awareness of sex‐stratified clinical presentations in autism, this study identified early‐life physical illnesses in autistic young children with and without intellectual disabilities. The sex‐differential findings implicate that sex‐aware and intellectual disabilities‐aware and tailored screening and health promotion strategies, such as monitoring and preventing for cardiovascular and metabolic risks in autistic females without intellectual disabilities, and enhanced epilepsy surveillance in those with intellectual disabilities, can be further developed. Public health efforts should also incorporate sex‐ and cognition‐informed approaches to promote early detection and targeted intervention. Future research should investigate genetic and environmental mechanisms underlying these disparities to inform precision care in autism.

## AUTHOR CONTRIBUTIONS

**Yu‐Chieh Chuang**: Conceptualization; methodology; writing—original draft; writing—review and editing. **Yu‐Jui Huang**: Methodology; conceptualization; writing—original draft. **Meng‐Chuan Lai**: Writing—original draft; writing—review and editing; conceptualization; methodology; supervision; investigation. **Wen‐Yin Chen**: Funding acquisition; project administration. **Chun‐Hung Pan**: Funding acquisition; project administration. **Sheng‐Siang Su**: Methodology; formal analysis; data curation; investigation. **Chian‐Jue Kuo**: Writing—original draft; supervision; project administration; investigation; methodology; conceptualization; writing—review and editing.

## CONFLICT OF INTEREST STATEMENT

The authors declare no Conflicts of interest.

## ETHICAL CONSIDERATIONS

This study was approved by the Research Ethics Committee of Taipei City Hospital, Taiwan (approval number: TCHIRB‐11206009‐E; the date of approval: June 19, 2023). Due to the retrospective design and deidentified nature of the data, the ethics committee exempted the study from requiring informed consent.

## Supporting information

Supporting Information S1

## Data Availability

Data sharing is not applicable to this article as the national registry raw data is not accessible due to privacy protection regulations.
