# Supplementary material for: Sex differences in 5‐year incidence and prevalence of physical illnesses following early childhood autism diagnosis
Source: JCPP Adv. 2026 Feb 19:e70108. Online ahead of print. doi: 10.1002/jcv2.70108 (PMC13339513; doi:10.1002/jcv2.70108)
Supplement: Supplementary file 1 — Supporting Information S1 [file JCV2-9999-e70108-s001.docx]

**Sex Differences in 5-Year Incidence and Prevalence of Physical Illnesses Following Early Childhood Autism Diagnosis**

**Supporting Information**

**List of Supporting Figures and Tables**

**Table S1.** Diagnostic codes for various illnesses

**Table S2.** 5-year incidence of co-occurring physical illnesses in female versus male children in the general population (age- and sex-matched 1:20 to the autistic cohort, n=913,600, without excluding any individual with a pre-existing physical illness)

**Table S3.** 5-year incidence of co-occurring physical illnesses in autistic female versus male children (with unadjusted HR)

**Table S4.** Healthcare utilization follow-up for 5 years after the index date for autistic female and male children without intellectual disabilities

**Table S5.** 5-year incidence of co-occurring physical illnesses in autistic female versus male children without intellectual disabilities

**Table S6.** Healthcare utilization follow-up for 5 years after the index date for autistic female and male children with intellectual disabilities

**Table S7.** 5-year incidence of co-occurring physical illnesses in autistic female versus male children with intellectual disabilities

**Figure S1.** Adjusted hazard ratio (aHR) of newly developed physical illnesses within 5 years after an initial ASD diagnosis in autistic females compared to autistic males, without intellectual disabilities.

**Figure S2.** Adjusted hazard ratio (aHR) of newly developed physical illnesses within 5 years after an initial ASD diagnosis in autistic females compared to autistic males, with intellectual disabilities.

**Table S8.** 5-year incidence of co-occurring physical illnesses in autistic female versus male children, with index date of birth date

**Table S9.** 5-year incidence of co-occurring physical illnesses in autistic female versus male children, with the entire available follow-up period

**Table S10A.** Demographic and clinical characteristics of autistic female and male children at the index date (2001-2009)

**Table S10B.** Demographic and clinical characteristics of autistic female and male children at the index date (2010-2019)

**Table S1.** Diagnostic codes for various illnesses

| Physical illness | ICD-9-CM codes | ICD-10-CM codes |
| --- | --- | --- |
| **Cardiovascular diseases** |  |  |
| Hypertension | 401–405 | I10, I110, I119, I129, I120, I1310, I130, I1311, I132 |
| Ischaemic heart disease | 410–414 | I2109, R0989, I2101 , I2102 , I2109, R0989, I220, I2101, I2102, I2119, I221, I2111, I2129, I228, I214, I222, I2121, I213, I229, I241, I200, I240, I248, I249, I252, I208, I201, I209, I2582, I2583, I2510, I25110, I25111, I25118, I25119, I25700, I25701, I25708, I25709, I25710, I25711, I25718, I25719, I25720, I25721, I25728, I25729, I25730, I25731, I25738, I25739, I25750, I25751, I25758, I25759, I25760, I25761, I25768, I25769, I25790, I25791, I25798, I25799, I25810, I25811, I25812, I253, I2541, I2542, I255, I256, I2589, I259 |
| Other forms of heart disease | 420–429 | I301, I309, I300, I308, I330, I339, I409, I400, I401, I408, I312, I310, I311, I318, I313, I314, I319, I340, I341, I342, I348, I349, I350, I351, I352, I358, I359, I360, I361, I362, I368, I369, I370, I371, I372, I378, I379, I423, I421, I428, I424, I420, I422, I425, I429, I426, I427, I442, I443, I440, I441, I444, I445, I446, I447, I450, I451, I454, I452, I453, I455, I456, I458, I459, I471, I492, I470, I472, I493, I479, I480, I482, I489, I481, I483, I484, I490, I462, I468, I469, I494, I491, I495, R001, I498, I499, I502, I503, I504, I509, I501, I514, I32, I41, I38, I43, I39 |
| Congestive heart failure | 428, 429.3, 402.01, 402.11, 402.91, 404.01, 404.03, 404.11, 404.13, 404.91, 404.93 | I110, I130, I132, I420, I421, I422, I423, I424, I425, I426, I427, I428, I429, I501, I509, I517' I5020, I5021, I5022, I5023, I5030, I5031, I5032, I5033, I5040, I5041, I5042, I5043, I43 |
| **Cerebrovascular diseases** | 430–438 | I60-I69, G45-G46 |
| **Epilepsy** | 345 | G40 |
| **Respiratory diseases** |  |  |
| URI | 460 | J00 |
| Pneumonia | 480–486, 507 | J14, J17, J13, J120, J121, J122, J123, J128, J128, J129, J181, J150, J151, J154, J154, J153, J154, J152, J152, J152, J158, J155, J156, A481, J158, J159, J157, J160, J168, B250, A379, A221, B440, J180, J188, J189, J690, J691, J698 |
| Asthma | 493 | J452, J453, J454, J455, J459, J449, J440 |
| Chronic bronchitis | 491 | J42, J410, J411, J418, J449, J441, J440 |
| COPD | 490, 491, 492, 496, A323, A325 | J449, J440, J441 , J430, J431, J432, J438, J439 |
| **Gastrointestinal diseases** |  |  |
| Chronic hepatic disease | 571 | K70, K73, K75, K74, K76, R16 |
| Ulcer | 531–534 | K25, K31, K56, K26, K27, K28 |
| **Endocrine diseases** |  |  |
| Diabetes mellitus | 250 | E8-E11, E13 |
| **Other** |  |  |
| Diseases of arteries, arterioles, and capillaries | 440–449 | I700, I701, I758, I702, I709, I750, I703, I706, I707, I704, I705, I708, I710, I711, I712, I713, I714, I718, I715, I716, I719, I778, I790, I721, I722, I777, I723, I724, I720, I728, I729, I730, I731, I791, I798, I738, I739, I740, I741, I742, I743, I744, I745, I748, I749, M300, M302, M308, M317, M303, M310, M312, M301, M313, M315, M316, M311, M314, I770, I771, I772, I773, I774, I775, M318, M319, I776, I779, I780, I781, I788, I789 |
| Diseases of veins and lymphatics, and other diseases of circulatory system | 451–459 | I800, I801, I802, I803, I808, I809, I81, I820, I821, I822, I823, I824, I825, I826, I827, I828, I829, I82A, I82B, I82C, I830, I831, I832, I838, I839, K640, K641, K642, K643, K648, K644, K645, K649, I850, I851, I860, I861, I862, I863, I864, I868, I972, I890, I891, I898, I899, I951, I950, I958, I952, I953, I959, R58 |
| Cancer | 140–209 | C01, C07, C12, C19, C20, C23, C33, C37, C55, C58, C52, C61, C73, D45, C000, Z511, C001, C003, C004, C005, C006, C008, C002, C009, C020, C021, C022, C023, C028, C024, C029, C080, C081, C089, C030, C031, C039, C040, C041, C048, C049, C060, C061, C050, C051, C052, C058, C059, C062, C068, C069, C098, C099, C090, C091, C100, C101, C108, C102, C103, C104, C109, C110, C111, C112, C113, C118, C119, C130, C131, C132, C138, C139, C140, C142, C148, C153, C154, C155, C158, C159, C160, C7A0, C164, C163, C161, C162, C165, C166, C168, C169, C170, C171, C172, C173, C178, C179, C183, C184, C186, C187, C180, C181, C182, C185, C188, C189, C211, C210, C212, C218, C220, C222, C223, C224, C227, C228, C221, C229, C240, C241, C248, C249, C250, C251, C252, C253, C254, C257, C258, C259, C480, C451, C481, C488, C482, C260, C261, C269, C300, C301, C310, C311, C312, C313, C318, C319, C320, C321, C322, C323, C328, C329, C340, C341, C342, C343, C348, C349, C384, C450, C380, C452, C381, C382, C388, C383, C390, C399, C410, C411, C412, C413, C400, C401, C414, C402, C403, C408, C409, C419, C470, C490, C471, C491, C472, C492, C473, C493, C474, C494, C475, C495, C476, C496, C478, C498, C479, C499, C430, D030, C431, D031, C432, D032, C433, D033, C434, D034, C435, D035, C436, D036, C437, D037, C438, D038, C439, D039, C440, C4A0, C441, C4A1, C442, C4A2, C443, C4A3, C444, C4A4, C445, C4A5, C446, C4A6, C447, C4A7, C448, C4A8, C449, C4A9, C500, C501, C502, C503, C504, C505, C506, C508, C509, C460, C461, C462, C464, C465, C463, C467, C469, C530, C531, C538, C539, C58, C541, C542, C543, C549, C540, C548, C561, C562, C569, C570, C571, C573, C572, C574, C510, C511, C512, C519, C518, C577, C578, C579, C620, C621, C629, C600, C601, C602, C609, C630, C631, C632, C608, C637, C638, C639, C670, C671, C672, C673, C674, C675, C676, C677, C678, C679, C641, C642, C649, C651, C652, C659, C661, C662, C669, C680, C681, C688, C689, C694, C696, C695, C690, C691, C692, C693, C698, C699, C710, C711, C712, C713, C714, C715, C716, C717, C719, C722, C723, C724, C725, C700, C709, C720, C721, C701, C729, E312, C740, C741, C749, C750, C751, C752, C753, C754, C755, C758, C759, C760, C761, C762, C763, C764, C765, C457, C768, C770, C7B0, C771, C772, C773, C774, C775, C778, C779, C780, C781, C782, J910, C783, C784, C785, C786, R180, C787, C788, C790, C791, C792, C793, C794, C795, C796, C797, C798, C799, C7A1, C7A8, C7B1, C7B8, C800, D3A8, C459, C801, C833, C846, C847, C852, C964, C965, C835, C837, C830, C838, C839, C865, C866, C817, C819, C810, C814, C811, C812, C813, C820, C821, C822, C823, C824, C825, C826, C828, C829, C840, C844, C849, C84A, C84Z, C860, C862, C863, C861, C841, C96A, C914, C960, C962, C831, C851, C858, C859, C864, C884, C969, C96Z, C900, C901, C882, C883, C888, C889, C902, C903, C910, C911, C91Z, C913, C915, C916, C91A, C919, C920, C924, C925, C926, C92A, C921, C922, C923, C92Z, C929, C933, C930, C931, C939, C93Z, C940, C942, C943, C948, C950 |
| Connective tissue disease | 710, 714 | M32, M34, M35, M33, M36, M05, M06, M08, M12 |
| Renal failure | 585, 586, V56, V42.0, V45.1, 39.27, 39.42, 39.93, 39.94, 39.95, 54.98 | N184, N185, N186, N189 Procedures code (0313, 0314, 0315, 0316, 0317, 0318, 0319, 031A, 031B, 031C, 0312, 03WY, 03PY, 5A1D, 3E1M) |
| HIV infection | 042, 043, 044 | B20 |
| Atopic dermatitis and related conditions | 691 | L22, L200, L208, L209 |
| Irritable bowel syndrome | 564.1 | K580, K589 |
| Hyperlipidemia | 272 | E780, E781, E782, E783, E784, E785, E786, E881, E752, E753, E770, E771, E778, E779, E713, E755, E787, E788, E882, E888, E756, E789 |
| **Intellectual disabilities** |  |  |
| Mild | 317 | F70 |
| Moderate | 318.0 | F71 |
| >Severe | 318.1, 318.2 | F72-F73 |
| Other/unspecified | 319 | F78-F79 |
| **Congenital deformities, chromosome abnormalities** | 740-759 | Q00-Q99 |

**Abbreviations:** COPD, chronic obstructive pulmonary disease; URI, upper respiratory tract infection.

The diseases were diagnosed using the *International Classification of Diseases*, *Ninth Revision*, *Clinical Modification* and *International Classification of Diseases*, *Tenth Revision*, *Clinical Modification* codes.

**Table S2.** 5-year incidence of co-occurring physical illnesses in female versus male children in the general population (age- and sex-matched 1:20 to the autistic cohort, n=913,600, without excluding any individual with a pre-existing physical illness)

| Characteristics | Females | Males | Total N | Adjusted HR^a^ | 95% confidence interval | P-value |
| --- | --- | --- | --- | --- | --- | --- |
| **5-year after the baseline** |  |  |  |  |  |  |
| **Physical illnesses** |  |  |  |  |  |  |
| Cardiovascular diseases |  |  |  |  |  |  |
| Hypertension | 159 (0.09) | 739 (0.10) | 913,012 | 0.96 | 0.81-1.14 | 0.671 |
| Ischemic heart disease | 138 (0.08) | 555 (0.08) | 912,700 | 1.09 | 0.91-1.32 | 0.358 |
| Other forms of heart disease | 2,009 (1.13) | 8,578 (1.19) | 901,058 | 1.04 | 0.99-1.09 | 0.115 |
| Congestive heart failure | 139 (0.08) | 643 (0.09) | 911,188 | 0.96 | 0.80-1.15 | 0.658 |
| Cerebrovascular diseases | 152 (0.08) | 1,024 (0.14) | 911,610 | 0.66 | 0.55-0.78 | <.001 |
| Epilepsy | 785 (0.44) | 4,196 (0.58) | 905,759 | 0.82 | 0.76-0.88 | <.001 |
| Respiratory diseases |  |  |  |  |  |  |
| Pneumonia | 36,889 (29.17) | 142,316 (29.72) | 605,358 | 1.02 | 1.00-1.03 | 0.014 |
| COPD | 5,723 (3.30) | 29,031 (4.17) | 869,273 | 0.86 | 0.83-0.88 | <.001 |
| Chronic bronchitis | 2,250 (1.26) | 11,348 (1.57) | 903,884 | 0.88 | 0.84-0.92 | <.001 |
| Asthma | 31,789 (20.50) | 147,066 (24.88) | 746,109 | 0.85 | 0.84-0.86 | <.001 |
| Upper respiratory tract infection | 34,932 (46.17) | 135,086 (47.05) | 362,757 | 1.01 | 1.00-1.02 | 0.042 |
| Gastrointestinal diseases |  |  |  |  |  |  |
| Chronic hepatic disease | 428 (0.24) | 1,719 (0.24) | 910,168 | 1.07 | 0.96-1.19 | 0.238 |
| Ulcer disease | 1,246 (0.69) | 5,688 (0.78) | 909,055 | 0.98 | 0.93-1.05 | 0.611 |
| Endocrine diseases |  |  |  |  |  |  |
| Diabetes mellitus | 168 (0.09) | 666 (0.09) | 913,109 | 1.13 | 0.95-1.33 | 0.168 |
| Others |  |  |  |  |  |  |
| Diseases of arteries, arterioles, and capillaries | 677 (0.38) | 3,074 (0.42) | 907,451 | 0.93 | 0.86-1.02 | 0.108 |
| Diseases of veins and lymphatics, and other diseases of circulatory System | 491 (0.27) | 2,361 (0.32) | 910,506 | 0.92 | 0.84-1.02 | 0.112 |
| Cancer | 435 (0.24) | 2,298 (0.31) | 911,530 | 0.82 | 0.74-0.91 | <.001 |
| Connective tissue disease | 293 (0.16) | 1,072 (0.15) | 912,912 | 1.23 | 1.08-1.40 | 0.002 |
| Renal failure | 41 (0.02) | 113 (0.02) | 913,374 | 1.62 | 1.13-2.32 | 0.008 |
| HIV infection | 3 (0.00) | 20 (0.00) | 913,544 | 0.66 | 0.20-2.25 | 0.512 |
| Atopic dermatitis and related conditions | 14,042 (13.87) | 51,417 (12.85) | 501,514 | 1.09 | 1.07-1.11 | <.001 |
| Irritable bowel syndrome | 1,278 (0.72) | 5,603 (0.77) | 901,946 | 0.99 | 0.93-1.05 | 0.656 |
| Hyperlipidemia | 190 (0.11) | 479 (0.07) | 913,393 | 1.82 | 1.54-2.15 | <.001 |
| **Neurodevelopmental disorder** |  |  |  |  |  |  |
| Intellectual disabilities |  |  |  |  |  |  |
| Mild | 408 (0.23) | 3,100 (0.42) | 913,106 | 0.60 | 0.55-0.67 | <.001 |
| Moderate | 184 (0.10) | 965 (0.13) | 913,435 | 0.90 | 0.77-1.05 | 0.182 |
| >Severe | 51 (0.03) | 234 (0.03) | 913,534 | 1.05 | 0.76-1.45 | 0.780 |
| Other/unspecified | 532 (0.29) | 3,562 (0.49) | 912,772 | 0.69 | 0.63-0.75 | <.001 |
| **Congenital deformities, chromosome abnormalities** | 4,055 (2.55) | 23,544 (3.74) | 788,184 | 0.71 | 0.68-0.73 | <.001 |

**Table S3.** 5-year incidence of co-occurring physical illnesses in autistic female versus male children (with unadjusted HR)

| Characteristics | Females | Males | Total N | Unadjusted HR | 95% confidence intervals | P-value |  |
| --- | --- | --- | --- | --- | --- | --- | --- |
| **5-year after the baseline** |  |  |  |  |  |  |  |
| **Physical illnesses** |  |  |  |  |  |  |  |
| Cardiovascular diseases |  |  |  |  |  |  |  |
| Hypertension | 15 (0.17) | 63 (0.17) | 45,628 | 1.01 | 0.58-1.78 | 0.961 |  |
| Ischemic heart disease | 14 (0.16) | 51 (0.14) | 45,608 | 1.16 | 0.64-2.10 | 0.626 |  |
| Other forms of heart disease | 178 (2.04) | 551 (1.54) | 44,554 | 1.40 | 1.18-1.65 | <.001 |  |
| Congestive heart failure | 29 (0.33) | 47 (0.13) | 45,362 | 2.60 | 1.64-4.13 | <.001 |  |
|  |  |  |  |  |  |  |  |
| Cerebrovascular diseases | 105 (1.18) | 329 (0.91) | 45,122 | 1.34 | 1.08-1.67 | 0.009 |  |
| Epilepsy | 410 (4.94) | 1,077 (3.06) | 43,522 | 1.70 | 1.52-1.90 | <.001 |  |
| Respiratory diseases |  |  |  |  |  |  |  |
| Pneumonia | 1,863 (33.87) | 7,171 (33.30) | 27,035 | 1.04 | 0.99-1.10 | 0.108 |  |
| COPD | 330 (3.91) | 1,842 (5.41) | 42,483 | 0.74 | 0.66-0.83 | 0.738 |  |
| Chronic bronchitis | 160 (1.80) | 742 (2.06) | 44,945 | 0.90 | 0.76-1.07 | 0.244 |  |
| Asthma | 1,654 (22.53) | 7,553 (27.16) | 35,149 | 0.82 | 0.78-0.87 | <.001 |  |
| Upper respiratory tract infection | 1,631 (49.25) | 6,392 (49.73) | 16,166 | 1.02 | 0.97-1.08 | 0.455 |  |
| Gastrointestinal diseases |  |  |  |  |  |  |  |
| Chronic hepatic disease | 42 (0.47) | 138 (0.38) | 45,360 | 1.29 | 0.92-1.82 | 0.145 |  |
| Ulcer disease | 101 (1.13) | 360 (0.99) | 45,292 | 1.19 | 0.95-1.48 | 0.135 |  |
| Endocrine diseases |  |  |  |  |  |  |  |
| Diabetes mellitus | 45 (0.50) | 289 (0.79) | 45,508 | 0.65 | 0.48-0.89 | 0.007 |  |
| Others |  |  |  |  |  |  |  |
| Diseases of arteries, arterioles, and capillaries | 40 (0.45) | 173 (0.48) | 45,250 | 0.97 | 0.69-1.37 | 0.875 |  |
| Diseases of veins and lymphatics, and other diseases of circulatory system | 42 (0.47) | 154 (0.42) | 45,401 | 1.16 | 0.82-1.63 | 0.398 |  |
| Cancer | 79 (0.88) | 300 (0.83) | 45,248 | 1.10 | 0.86-1.41 | 0.433 |  |
| Connective tissue disease | 32 (0.36) | 88 (0.24) | 45,621 | 1.55 | 1.03-2.33 | 0.034 |  |
| Renal failure | - | - | 45,648 | - | - | - |  |
| HIV infection | - | - | 45,677 | - | - | - |  |
| Atopic dermatitis and related conditions | 700 (15.59) | 2,666 (15.00) | 22,262 | 1.07 | 0.99-1.17 | 0.092 |  |
| Irritable bowel syndrome | 108 (1.22) | 445 (1.24) | 44,748 | 1.03 | 0.83-1.27 | 0.801 |  |
| Hyperlipidemia | 27 (0.30) | 61 (0.17) | 45,656 | 1.91 | 1.21-3.00 | 0.005 |  |
| **Neurodevelopmental disorder** |  |  |  |  |  |  |  |
| Intellectual disabilities |  |  |  |  |  |  |  |
| Mild | 737 (8.43) | 2,785 (7.76) | 44,617 | 1.14 | 1.05-1.23 | 0.002 |  |
| Moderate | 523 (5.91) | 1,731 (4.78) | 45,069 | 1.30 | 1.18-1.44 | <.001 |  |
| >Severe | 206 (2.30) | 481 (1.32) | 45,525 | 1.85 | 1.57-2.18 | <.001 |  |
| Other/unspecified | 850 (9.89) | 2,784 (7.86) | 44,028 | 1.33 | 1.23-1.43 | <.001 |  |
| **Congenital deformities, chromosome abnormalities** | 452 (6.72) | 1,990 (7.05) | 34,951 | 0.98 | 0.89-1.09 | 0.724 |  |

**Table S4.** Healthcare utilization follow-up for 5 years after the index date for autistic female and male children without intellectual disabilities

| Characteristic N (%) | Females  (N=6,723) | Males  (N=29,021) | t value | P-value |
| --- | --- | --- | --- | --- |
|  | Mean (SD) | Mean (SD) |  |  |
|  |  |  |  |  |
| Number of hospital admissions, *mean (SD)* | 0.76 (1.95) | 0.64 (1.84) | 4.52 | <.001 |
| non-psychiatric hospital admissions | 0.72 (1.92) | 0.58 (1.80) | 5.44 | <.001 |
| psychiatric hospital admissions | 0.04 (0.27) | 0.06 (0.33) | -5.68 | <.001 |
| Number of outpatient visits, *mean (SD)* | 138.30 (80.48) | 147.70 (82.44) | -8.61 | <.001 |
| non-psychiatric outpatient visits | 132.00 (77.68) | 138.60 (78.90) | -6.13 | <.001 |
| psychiatric outpatient visits | 6.27 (11.78) | 9.16 (13.84) | -17.50 | <.001 |
|  |  |  |  |  |
| *Specialist (number of outpatient visits)* |  |  |  |  |
| General medicine | 1.22 (6.78) | 1.66 (8.44) | -4.51 | <.001 |
| Family practice | 9.14 (17.62) | 9.62 (18.99) | -1.99 | 0.047 |
| Internal medicine | 3.12 (9.36) | 3.28 (10.20) | -1.20 | 0.229 |
| Surgery | 0.70 (3.20) | 0.75 (2.64) | -1.18 | 0.237 |
| Pediatrics | 38.40 (36.58) | 39.12 (38.04) | -1.43 | 0.153 |
| Gynecology | 0.19 (1.12) | 0.07 (1.23) | 8.30 | <.001 |
| Orthopedics | 0.56 (1.77) | 0.59 (1.70) | -1.19 | 0.235 |
| Neurosurgery | 0.06 (0.63) | 0.05 (0.45) | 1.67 | 0.096 |
| Urology | 0.06 (0.47) | 0.25 (0.98) | -23.42 | <.001 |
| Otorhinolaryngology | 16.85 (25.74) | 18.82 (28.24) | -5.54 | <.001 |
| Ophthalmology | 6.78 (7.89) | 6.75 (7.57) | 0.20 | 0.839 |
| Dermatology | 2.10 (4.38) | 2.01 (4.39) | 1.45 | 0.146 |
| Neurology | 0.09 (1.40) | 0.06 (1.09) | 1.52 | 0.129 |
| Psychiatry | 6.27 (11.78) | 9.16 (13.84) | -17.50 | <.001 |
| Rehabilitation | 32.44 (39.78) | 34.92 (41.82) | -4.56 | <.001 |
| Plastic Surgery | 0.13 (0.89) | 0.13 (0.81) | 0.25 | 0.805 |
| Emergent department | 1.62 (2.72) | 1.84 (2.94) | -5.84 | <.001 |
| Occupational medicine | 0.00 (0.02) | 0.00 (0.03) | -0.55 | 0.585 |
| Tuberculosis department | 0.00 (0.15) | 0.00 (0.14) | 0.11 | 0.912 |
| Hemodialysis department | 0.00 (0.00) | 0.00 (0.29) | -1.00 | 0.317 |
| Dentistry | 11.71 (8.09) | 11.58 (8.10) | 1.15 | 0.249 |
| Chinese herb medicine | 6.83 (18.66) | 7.05 (18.49) | -0.89 | 0.373 |
| Anesthesiology | 0.00 (0.03) | 0.00 (0.02) | 1.05 | 0.292 |
| Radiology | 0.01 (0.12) | 0.01 (0.18) | 2.38 | 0.018 |
| Pathology | 0.00 (0.04) | 0.00 (0.05) | -0.63 | 0.530 |

**Table S5.** 5-year incidence of co-occurring physical illnesses in autistic female versus male children without intellectual disabilities

| Characteristics | Females  (N=6,723) | Males  (N=29,021) | Total N | adjusted HR ^a^ | 95% confidence intervals | P-value |
| --- | --- | --- | --- | --- | --- | --- |
| **5-year after the baseline** |  |  |  |  |  |  |
| **Physical illnesses** |  |  |  |  |  |  |
| Cardiovascular diseases |  |  |  |  |  |  |
| Hypertension | 9 (0.13) | 46 (0.16) | 35,708 | 0.93 | 0.46-1.87 | 0.840 |
| Ischemic heart disease | 10 (0.15) | 37 (0.13) | 35,686 | 1.29 | 0.64-2.61 | 0.480 |
| Other forms of heart disease | 120 (1.84) | 395 (1.39) | 34,949 | 1.44 | 1.18-1.77 | <.001 |
| Congestive heart failure | 18 (0.27) | 28 (0.10) | 35,541 | 2.86 | 1.62-5.06 | <.001 |
| Cerebrovascular diseases | 52 (0.78) | 205 (0.71) | 35,385 | 1.15 | 0.85-1.56 | 0.375 |
| Epilepsy | 196 (3.06) | 606 (2.14) | 34,660 | 1.55 | 1.32-1.82 | <.001 |
| Respiratory diseases |  |  |  |  |  |  |
| Pneumonia | 1,400 (33.40) | 5,695 (33.22) | 21,336 | 1.04 | 0.98-1.10 | 0.191 |
| COPD | 228 (3.58) | 1366 (5.05) | 33,399 | 0.76 | 0.66-0.87 | <.001 |
| Chronic bronchitis | 122 (1.84) | 570 (2.00) | 35,175 | 1.01 | 0.83-1.23 | 0.898 |
| Asthma | 1258 (22.94) | 6065 (27.64) | 27,423 | 0.85 | 0.80-0.90 | <.001 |
| Upper respiratory tract infection | 1,232 (49.14) | 4,963 (48.67) | 12,704 | 1.06 | 1.00-1.13 | 0.053 |
| Gastrointestinal diseases |  |  |  |  |  |  |
| Chronic hepatic disease | 30 (0.45) | 92 (0.32) | 35,508 | 1.49 | 0.99-2.23 | 0.057 |
| Ulcer disease | 65 (0.97) | 254 (0.88) | 35,482 | 1.24 | 0.94-1.63 | 0.132 |
| Endocrine diseases |  |  |  |  |  |  |
| Diabetes mellitus | 24 (0.36) | 201 (0.69) | 35,617 | 0.56 | 0.36-0.85 | 0.007 |
| Others |  |  |  |  |  |  |
| Diseases of arteries, arterioles, and capillaries | 28 (0.42) | 142 (0.49) | 35,397 | 0.91 | 0.60-1.36 | 0.628 |
| Diseases of veins and lymphatics, and other Diseases of circulatory system | 22 (0.33) | 97 (0.34) | 35,542 | 1.08 | 0.68-1.71 | 0.751 |
| Cancer | 47 (0.71) | 235 (0.82) | 35,420 | 0.92 | 0.67-1.26 | 0.596 |
| Connective tissue disease | 21 (0.31) | 70 (0.24) | 35,701 | 1.49 | 0.91-2.45 | 0.116 |
| Renal failure | - | - | 35,724 | - | - | - |
| HIV infection | - | - | - | - | - | - |
| Atopic dermatitis and related conditions | 523 (16.02) | 2,007 (14.73) | 16,892 | 1.14 | 1.03-1.25 | 0.009 |
| Irritable bowel syndrome | 81 (1.23) | 314 (1.10) | 35,045 | 1.25 | 0.98-1.60 | 0.076 |
| Hyperlipidemia | 18 (0.27) | 42 (0.14) | 35,731 | 2.11 | 1.21-3.68 | 0.008 |
| **Congenital deformities, chromosome abnormalities** | - | - | - | 0.90 | 0.79-1.03 | 0.117 |

^a^ Adjusted with demographic and clinical characteristics (except for sex) in Table 1, and numbers of outpatient visits, hospital admissions in e-Table 3

**Table S6.** Healthcare utilization follow-up for 5 years after the index date for autistic female and male children with intellectual disabilities

| Characteristic N (%) | Females  (N=2,301) | Males  (N=7,365) | t | P-value |
| --- | --- | --- | --- | --- |
|  | Mean (SD) | Mean (SD) |  |  |
|  |  |  |  |  |
| Number of hospital admissions, *mean (SD)* | 1.25 (3.03) | 0.98 (2.09) | 3.94 | <.001 |
| non-psychiatric hospital admissions | 1.19 (3.00) | 0.90 (2.04) | 4.29 | <.001 |
| psychiatric hospital admissions | 0.07 (0.33) | 0.09 (0.38) | -2.42 | 0.016 |
| Number of outpatient visits, *mean (SD)* | 185.70 (100.30) | 188.50 (100.90) | -1.16 | 0.246 |
| non-psychiatric outpatient visits | 176.30 (97.82) | 176.70 (98.07) | -0.16 | 0.871 |
| psychiatric outpatient visits | 9.38 (13.17) | 11.78 (15.44) | -7.36 | <.001 |
|  |  |  |  |  |
| *Specialist (number of outpatient visits)* |  |  |  |  |
| General medicine | 2.82 (10.55) | 3.40 (12.35) | -2.21 | 0.027 |
| Family practice | 12.36 (23.61) | 11.84 (22.03) | 0.95 | 0.344 |
| Internal medicine | 4.27 (11.82) | 4.38 (11.53) | -0.39 | 0.696 |
| Surgery | 0.92 (2.80) | 1.11 (3.65) | -2.69 | 0.007 |
| Pediatrics | 44.86 (40.05) | 43.61 (42.71) | 1.30 | <.001 |
| Gynecology | 0.21 (1.20) | 0.12 (2.00) | 2.78 | 0.005 |
| Orthopedics | 0.75 (2.27) | 0.72 (2.87) | 0.49 | 0.626 |
| Neurosurgery | 0.12 (0.84) | 0.11 (0.86) | 0.40 | 0.686 |
| Urology | 0.19 (2.06) | 0.30 (1.24) | -2.47 | 0.014 |
| Otorhinolaryngology | 19.63 (29.85) | 22.07 (31.67) | -3.39 | <.001 |
| Ophthalmology | 6.39 (7.74) | 6.05 (7.18) | 1.92 | 0.055 |
| Dermatology | 2.04 (4.11) | 1.98 (4.26) | 0.62 | 0.532 |
| Neurology | 0.20 (1.98) | 0.14 (1.76) | 1.26 | 0.208 |
| Psychiatry | 9.38 (13.17) | 11.78 (15.44) | -7.36 | <.001 |
| Rehabilitation | 57.33 (53.16) | 58.65 (53.80) | -1.03 | 0.304 |
| Plastic Surgery | 0.21 (1.10) | 0.17 (0.98) | 1.93 | 0.053 |
| Emergent department | 2.15 (3.81) | 2.22 (3.84) | -0.85 | 0.397 |
| Occupational medicine | 0.00 (0.02) | 0.00 (0.01) | 0.67 | 0.504 |
| Tuberculosis department | 0.01 (0.24) | 0.01 (0.20) | 0.29 | 0.771 |
| Hemodialysis department | 0.00 (0.00) | 0.00 (0.09) | -1.00 | 0.317 |
| Dentistry | 11.28 (8.71) | 10.84 (8.41) | 2.11 | 0.035 |
| Chinese herb medicine | 10.54 (25.25) | 8.97 (22.83) | 2.67 | 0.008 |
| Anesthesiology | 0.00 (0.04) | 0.00 (0.04) | -0.72 | 0.473 |
| Radiology | 0.02 (0.16) | 0.01 (0.12) | 2.22 | 0.027 |
| Pathology | 0.00 (0.03) | 0.00 (0.05) | -0.68 | 0.496 |

**Table S7.** 5-year incidence of co-occurring physical illnesses in autistic female versus male children with intellectual disabilities

| Characteristics | Females  (N=2,301) | Males  (N=7,365) | Total N | adjusted HR ^a^ | 95% confidence intervals | P-value |
| --- | --- | --- | --- | --- | --- | --- |
| **5-year incidence after the baseline** |  |  |  |  |  |  |
| **Physical illnesses** |  |  |  |  |  |  |
| Cardiovascular diseases |  |  |  |  |  |  |
| Hypertension | 6 (0.26) | 17 (0.22) | 9920 | 1.17 | 0.45-3.06 | 0.748 |
| Ischemic heart disease | 4 (0.17) | 14 (0.18) | 9922 | 1.00 | 0.33-2.99 | 0.994 |
| Other forms of heart disease | 58 (2.63) | 156 (2.11) | 9605 | 1.19 | 0.87-1.63 | 0.273 |
| Congestive heart failure | 11 (0.49) | 19 (0.25) | 9821 | 1.78 | 0.82-3.86 | 0.142 |
| Cerebrovascular diseases | 53 (2.36) | 124 (1.65) | 9737 | 1.45 | 1.04-2.01 | 0.027 |
| Epilepsy | 214 (11.29) | 471 (6.76) | 8862 | 1.81 | 1.53-2.13 | <.001 |
| Respiratory diseases |  |  |  |  |  |  |
| Pneumonia | 463 (35.34) | 1476 (33.63) | 5699 | 1.01 | 0.90-1.12 | 0.931 |
| COPD | 102 (4.91) | 476 (6.79) | 9084 | 0.71 | 0.57-0.88 | 0.002 |
| Chronic bronchitis | 38 (1.68) | 172 (2.29) | 9770 | 0.74 | 0.52-1.07 | 0.109 |
| Asthma | 396 (21.34) | 1488 (25.35) | 7726 | 0.83 | 0.74-0.92 | <.001 |
| Upper respiratory tract infection | 399 (49.57) | 1429 (53.78) | 3462 | 0.92 | 0.83-1.03 | 0.166 |
| Gastrointestinal diseases |  |  |  |  |  |  |
| Chronic hepatic disease | 12 (0.53) | 46 (0.61) | 9852 | 0.81 | 0.42-1.56 | 0.536 |
| Ulcer disease | 36 (1.59) | 106 (1.40) | 9810 | 1.10 | 0.75-1.61 | 0.641 |
| Endocrine diseases |  |  |  |  |  |  |
| Diabetes mellitus | 21 (0.92) | 88 (1.16) | 9891 | 0.84 | 0.52-1.35 | 0.463 |
| Others |  |  |  |  |  |  |
| Diseases of arteries, arterioles, and capillaries | 12 (0.53) | 31 (0.41) | 9853 | 1.27 | 0.64-2.53 | 0.503 |
| Diseases of veins and lymphatics, and other Diseases of circulatory system | 20 (0.88) | 57 (0.75) | 9859 | 1.10 | 0.66-1.84 | 0.721 |
| Cancer | 32 (1.41) | 65 (0.86) | 9828 | 1.64 | 1.07-2.52 | 0.024 |
| Connective tissue disease | 11 (0.48) | 18 (0.24) | 9920 | 1.89 | 0.89-4.00 | 0.097 |
| Renal failure | - | - | - | - | - | - |
| HIV infection | - | - | - | - | - | - |
| Atopic dermatitis and related conditions | 177 (14.45) | 659 (15.90) | 5370 | 0.91 | 0.77-1.08 | 0.295 |
| Irritable bowel syndrome | 27 (1.20) | 131 (1.76) | 9703 | 0.69 | 0.45-1.06 | 0.093 |
| Hyperlipidemia | 9 (0.39) | 19 (0.25) | 9925 | 1.54 | 0.71-3.35 | 0.279 |
| **Congenital deformities, chromosome abnormalities** | 172 (10.96) | 550 (10.01) | 7066 | 1.12 | 0.94-1.33 | 0.212 |

^a^ Adjusted with demographic and clinical characteristics (except for sex) in Table 1, and numbers of outpatient visits, hospital admissions in e-Table 5

**Figure S1.** Adjusted hazard ratio (aHR) of newly developed physical illnesses within 5 years after an initial ASD diagnosis in autistic females compared to autistic males, without intellectual disabilities.

**
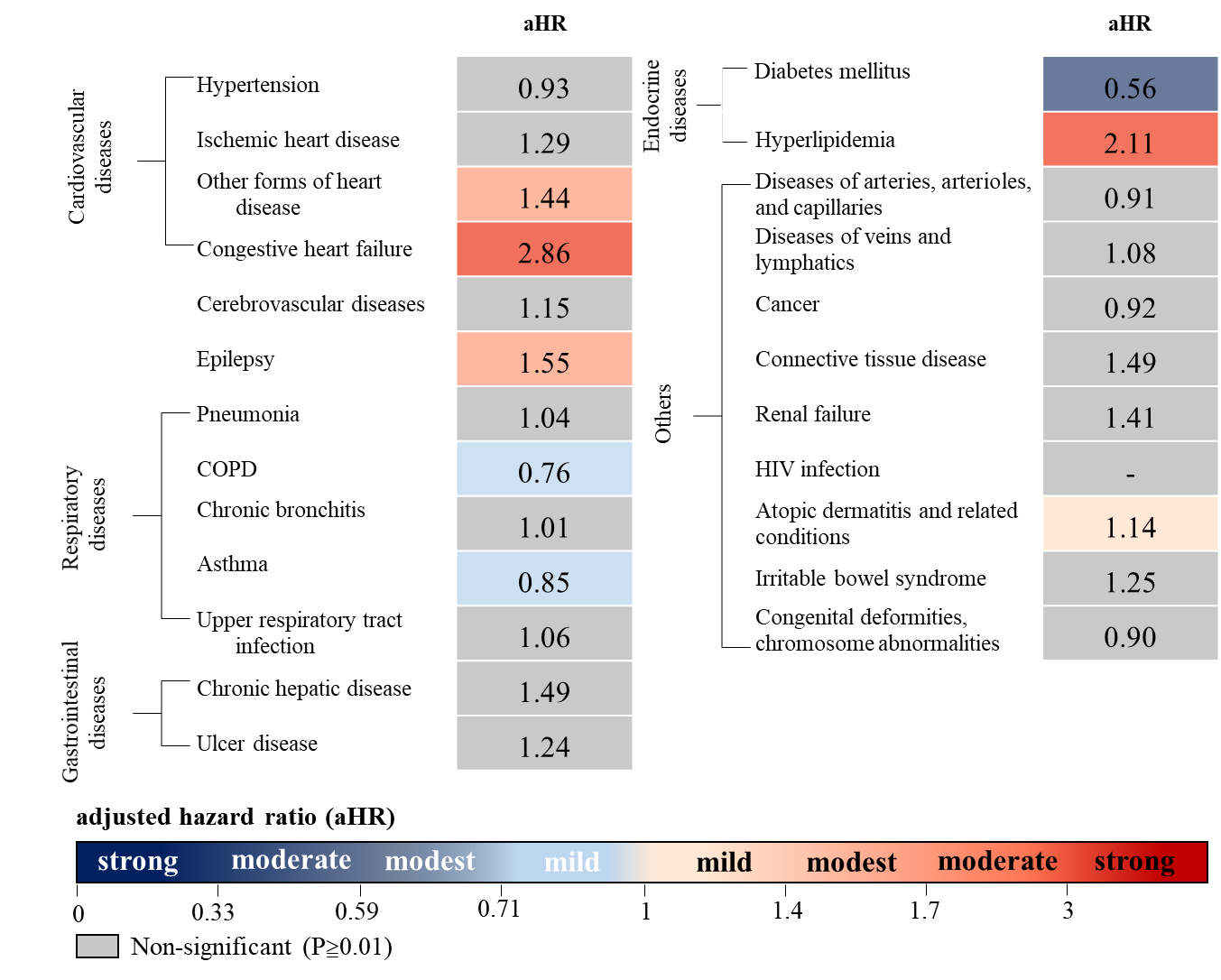
**

**Figure S2.** Adjusted hazard ratio (aHR) of newly developed physical illnesses within 5 years after an initial ASD diagnosis in autistic females compared to autistic males, with intellectual disabilities.

**
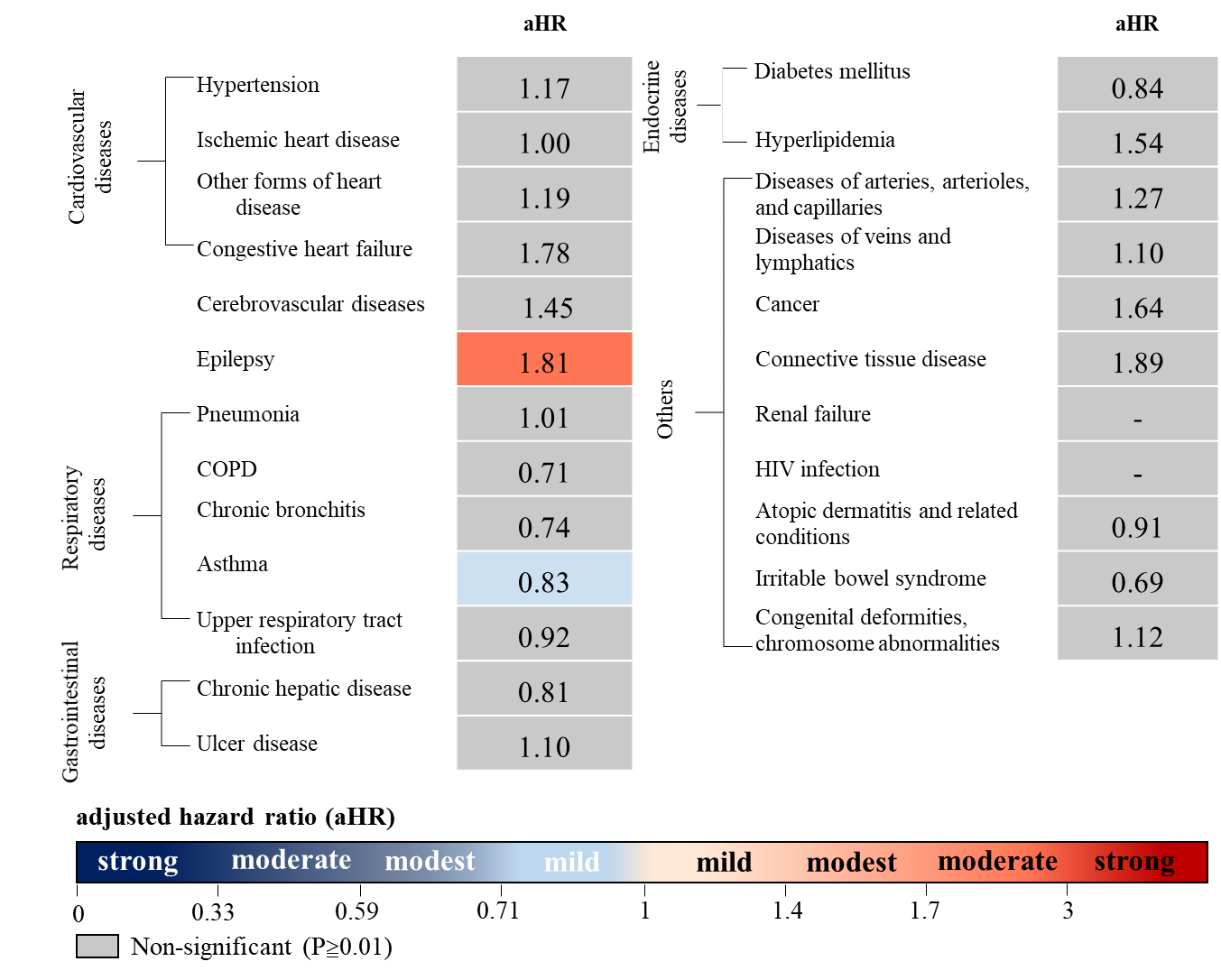
**

**Table S8.** 5-year incidence of co-occurring physical illnesses in autistic female versus male children, with index date of birth date

| Characteristics | Females | Males | Total N | Adjusted HR^a^ | 95% confidence interval | P-value |
| --- | --- | --- | --- | --- | --- | --- |
| **5-year after the baseline** |  |  |  |  |  |  |
| **Physical illnesses** |  |  |  |  |  |  |
| Cardiovascular diseases |  |  |  |  |  |  |
| Hypertension | 15 (0.17) | 58 (0.17) | 45,668 | 1.14 | 0.50-2.57 | 0.759 |
| Ischemic heart disease | 22 (0.24) | 70 (0.19) | 45,668 | 1.85 | 0.88-3.92 | 0.107 |
| Other forms of heart disease | 266 (2.99) | 681 (1.87) | 45,239 | 1.98 | 1.54-2.56 | <.001 |
| Congestive heart failure | 67 (0.75) | 127 (0.35) | 45,513 | 4.02 | 2.21-7.31 | <.001 |
| Cerebrovascular diseases | 185 (2.06) | 577 (1.58) | 45,582 | 1.29 | 1.00-1.66 | 0.048 |
| Epilepsy | 828 (9.33) | 1726 (4.78) | 45,245 | 2.00 | 1.73-2.30 | <.001 |
| Respiratory diseases |  |  |  |  |  |  |
| Pneumonia | 4531 (52.78) | 18673 (53.71) | 43,354 | 1.03 | 0.97-1.09 | 0.286 |
| COPD | 715 (7.99) | 3,487 (9.59) | 45,326 | 0.71 | 0.61-0.82 | <.001 |
| Chronic bronchitis | 227 (2.52) | 982 (2.68) | 45,637 | 0.95 | 0.76-1.18 | 0.638 |
| Asthma | 2757 (30.86) | 13807 (38.21) | 45,069 | 0.80 | 0.75-0.85 | <.001 |
| Upper respiratory tract infection | 5492 (72.06) | 22339 (72.81) | 38,301 | 1.03 | 0.97-1.10 | 0.357 |
| Gastrointestinal diseases |  |  |  |  |  |  |
| Chronic hepatic disease | 80 (0.89) | 250 (0.68) | 45,604 | 1.30 | 0.81-2.09 | 0.282 |
| Ulcer disease | 112 (1.24) | 431 (1.18) | 45,629 | 0.99 | 0.70-1.39 | 0.944 |
| Endocrine diseases |  |  |  |  |  |  |
| Diabetes mellitus | 76 (0.84) | 325 (0.89) | 45,675 | 0.64 | 0.44-0.93 | 0.021 |
| Others |  |  |  |  |  |  |
| Diseases of arteries, arterioles, and capillaries | 76 (0.84) | 414 (1.13) | 45,623 | 0.98 | 0.62-1.55 | 0.936 |
| Diseases of veins and lymphatics, and other diseases of circulatory System | 79 (0.88) | 255 (0.70) | 45,628 | 1.10 | 0.69-1.77 | 0.695 |
| Cancer | 125 (1.39) | 486 (1.33) | 45,631 | 0.85 | 0.61-1.17 | 0.314 |
| Connective tissue disease | 22 (0.24) | 71 (0.19) | 45,667 | 0.76 | 0.34-1.74 | 0.522 |
| Renal failure | 6 (0.07) | 23 (0.06) | 45,667 | 0.43 | 0.05-3.50 | 0.433 |
| HIV infection | - | - | - | - | - | - |
| Atopic dermatitis and related conditions | 3390 (45.63) | 13320 (45.24) | 36,869 | 1.06 | 0.96-1.18 | 0.268 |
| Irritable bowel syndrome | 217 (2.41) | 802 (2.20) | 45,485 | 1.22 | 0.92-1.61 | 0.176 |
| Hyperlipidemia | 17 (0.19) | 24 (0.07) | 45,672 | 2.06 | 0.86-4.92 | 0.104 |
| **Neurodevelopmental disorder** |  |  |  |  |  |  |
| Intellectual disabilities |  |  |  |  |  |  |
| Mild | 577 (6.40) | 1899 (5.18) | 45,676 | 1.16 | 1.04-1.31 | 0.011 |
| Moderate | 407 (4.51) | 1192 (3.25) | 45,674 | 1.37 | 1.20-1.57 | <.001 |
| >Severe | 113 (1.25) | 268 (0.73) | 45,680 | 1.44 | 1.10-1.89 | 0.008 |
| Other/unspecified | 816 (9.05) | 2532 (6.91) | 45,667 | 1.29 | 1.16-1.42 | <.001 |
| **Congenital deformities, chromosome abnormalities** | 1506 (18.29) | 5689 (17.38) | 40,693 | 1.02 | 0.89-1.15 | 0.815 |

**Table S9.** 5-year incidence of co-occurring physical illnesses in autistic female versus male children, with the entire available follow-up period

| Characteristics | Females | Males | Total N | Adjusted HR^a^ | 95% confidence interval | P-value |
| --- | --- | --- | --- | --- | --- | --- |
| **5-year after the baseline** |  |  |  |  |  |  |
| **Physical illnesses** |  |  |  |  |  |  |
| Cardiovascular diseases |  |  |  |  |  |  |
| Hypertension | 34 (0.38) | 303 (0.83) | 45,628 | 0.49 | 0.35-0.70 | <.001 |
| Ischemic heart disease | 29 (0.32) | 119 (0.33) | 45,608 | 1.07 | 0.71-1.61 | 0.750 |
| Other forms of heart disease | 323 (3.70) | 1170 (3.27) | 44,554 | 1.24 | 1.09-1.40 | <.001 |
| Congestive heart failure | 38 (0.43) | 93 (0.26) | 45,362 | 1.75 | 1.20-2.55 | 0.004 |
| Cerebrovascular diseases | 141 (1.59) | 420 (1.16) | 45,122 | 1.45 | 1.20-1.76 | <.001 |
| Epilepsy | 546 (6.58) | 1652 (4.69) | 43,522 | 1.60 | 1.45-1.77 | <.001 |
| Respiratory diseases |  |  |  |  |  |  |
| Pneumonia | 2052 (37.30) | 8039 (37.33) | 27,035 | 1.04 | 0.99-1.09 | 0.152 |
| COPD | 420 (4.98) | 2,268 (6.66) | 42,483 | 0.78 | 0.71-0.87 | <.001 |
| Chronic bronchitis | 226 (2.54) | 1130 (3.13) | 44,945 | 0.87 | 0.76-1.01 | 0.059 |
| Asthma | 1834 (24.99) | 8459 (30.42) | 35,149 | 0.82 | 0.78-0.87 | <.001 |
| Upper respiratory tract infection | 1849 (55.83) | 7394 (57.52) | 16,166 | 1.02 | 0.97-1.07 | 0.498 |
| Gastrointestinal diseases |  |  |  |  |  |  |
| Chronic hepatic disease | 135 (1.51) | 807 (2.22) | 45,360 | 0.74 | 0.62-0.89 | 0.001 |
| Ulcer disease | 262 (2.93) | 1012 (2.78) | 45,292 | 1.14 | 1.00-1.31 | 0.054 |
| Endocrine diseases |  |  |  |  |  |  |
| Diabetes mellitus | 103 (1.15) | 506 (1.39) | 45,508 | 0.90 | 0.72-1.11 | 0.310 |
| Others |  |  |  |  |  |  |
| Diseases of arteries, arterioles, and capillaries | 77 (0.86) | 299 (0.82) | 45,250 | 1.13 | 0.88-1.45 | 0.342 |
| Diseases of veins and lymphatics, and other diseases of circulatory System | 101 (1.13) | 548 (1.50) | 45,401 | 0.83 | 0.67-1.02 | 0.078 |
| Cancer | 128 (1.43) | 451 (1.24) | 45,248 | 1.25 | 1.03-1.52 | 0.027 |
| Connective tissue disease | 80 (0.89) | 221 (0.60) | 45,621 | 1.62 | 1.25-2.09 | <.001 |
| Renal failure | 7 (0.08) | 49 (0.13) | 45,648 | 0.64 | 0.29-1.41 | 0.266 |
| HIV infection | - | 3 (0.01) | 45,677 | 0.00 | 0.00-0.00 | <.001 |
| Atopic dermatitis and related conditions | 903 (20.11) | 3601 (20.26) | 22,262 | 1.05 | 0.98-1.14 | 0.159 |
| Irritable bowel syndrome | 234 (2.65) | 960 (2.67) | 44,748 | 1.10 | 0.95-1.27 | 0.197 |
| Hyperlipidemia | 169 (1.87) | 692 (1.89) | 45,656 | 1.10 | 0.93-1.31 | 0.257 |
| **Neurodevelopmental disorder** |  |  |  |  |  |  |
| Intellectual disabilities |  |  |  |  |  |  |
| Mild | 980 (11.21) | 3726 (10.39) | 44,617 | 1.18 | 1.10-1.26 | <.001 |
| Moderate | 799 (9.03) | 2617 (7.22) | 45,069 | 1.40 | 1.29-1.52 | <.001 |
| >Severe | 371 (4.14) | 924 (2.53) | 45,525 | 1.85 | 1.64-2.09 | <.001 |
| Other/unspecified | 1124 (13.08) | 3849 (10.86) | 44,028 | 1.35 | 1.26-1.44 | <.001 |
| **Congenital deformities, chromosome abnormalities** | 585 (8.69) | 2649 (9.39) | 34,951 | 0.98 | 0.90-1.07 | 0.679 |

**Table S10A. Demographic and clinical characteristics of autistic female and male children at the index date (2001-2009)**

|  | Females  (N=2,452) | Males  (N=11,390) |  |
| --- | --- | --- | --- |
|  | *n* (%) | *n* (%) | P-value |
| Age, meanSD (years) | 3.07 (1.23) | 3.05 (1.22) | 0.364 |
| 1-year-old | 215 (8.77) | 939 (8.24) | 0.389 |
| 2-year-old | 709 (28.92) | 3506 (30.78) |  |
| 3-year-old | 613 (25.00) | 2846 (24.99) |  |
| 4-year-old | 510 (20.8) | 2254 (19.79) |  |
| 5-year-old | 405 (16.52) | 1845 (16.20) |  |
|  |  |  |  |
| Urbanization |  |  | 0.319 |
| Level 1 | 1298 (52.94) | 6151 (54.00) |  |
| Level 2 | 784 (31.97) | 3706 (32.54) |  |
| Level 3 | 69 (2.81) | 276 (2.42) |  |
| Level 4 | 130 (5.30) | 581 (5.10) |  |
| Level 5 | 161 (6.57) | 629 (5.52) |  |
| missing data | 10 (0.41) | 47 (0.41) |  |
| Parental monthly income, (TWD^*^) |  |  | <.001 |
| 0-20,479 (first quarter) | 722 (29.45) | 2923 (25.66) |  |
| 20,480-25,599 (second quarter) | 584 (23.82) | 2818 (24.74) |  |
| 25,600-44,799 (third quarter) | 579 (23.61) | 2705 (23.75) |  |
| ≧44,800 (fourth quarter) | 567 (23.12) | 2994 (25.85) |  |

*31 TWD (Taiwan dollar) ≈1 US dollar.

**Table S10B. Demographic and clinical characteristics of autistic female and male children at the index date (2010-2019)**

|  | Females  (N=6,572) | Males  (N=25,266) |  |
| --- | --- | --- | --- |
|  | *n* (%) | *n* (%) | P-value |
| Age, meanSD (years) | 2.99 (1.28) | 3.11 (1.25) | <.001 |
| 1-year-old | 836 (12.72) | 2243 (8.88) | <.001 |
| 2-year-old | 1827 (27.80) | 7142 (28.27) |  |
| 3-year-old | 1531 (23.30) | 6089 (24.10) |  |
| 4-year-old | 1326 (20.18) | 5242 (20.75) |  |
| 5-year-old | 1052 (16.01) | 4550 (18.01) |  |
|  |  |  |  |
| Urbanization |  |  | 0.143 |
| Level 1 | 3593 (54.67) | 13452 (53.24) |  |
| Level 2 | 2077 (31.60) | 8411 (33.29) |  |
| Level 3 | 151 (2.30) | 629 (2.49) |  |
| Level 4 | 315 (4.79) | 1168 (4.62) |  |
| Level 5 | 356 (5.42) | 1307 (5.17) |  |
| missing data | 80 (1.22) | 299 (1.18) |  |
| Parental monthly income, (TWD^*^) |  |  | <.001 |
| 0-20,479 (first quarter) | 1145 (17.42) | 3965 (15.69) |  |
| 20,480-25,599 (second quarter) | 1711 (26.03) | 6523 (25.82) |  |
| 25,600-44,799 (third quarter) | 2119 (32.24) | 7914 (31.32) |  |
| ≧44,800 (fourth quarter) | 1597 (24.30) | 6864 (27.17) |  |

*31 TWD (Taiwan dollar) ≈1 US dollar.
